# Neonatal imprinting of alveolar macrophages via neutrophil-derived 12-HETE

**DOI:** 10.1038/s41586-022-05660-7

**Published:** 2023-01-04

**Authors:** Erwan Pernet, Sarah Sun, Nicole Sarden, Saideep Gona, Angela Nguyen, Nargis Khan, Martin Mawhinney, Kim A. Tran, Julia Chronopoulos, Dnyandeo Amberkar, Mina Sadeghi, Alexandre Grant, Shradha Wali, Renaud Prevel, Jun Ding, James G. Martin, Ajitha Thanabalasuriar, Bryan G. Yipp, Luis B. Barreiro, Maziar Divangahi

**Affiliations:** 1 McGill University Health Centre, Meakins-Christie Laboratories, McGill University, Montreal, Quebec Canada; 2 Department of Medicine, Section of Genetic Medicine, University of Chicago, Chicago, IL USA; 3 Calvin, Phoebe and Joan Snyder Institute for Chronic Diseases and Department of Critical Care, Cumming School of Medicine, University of Calgary, Calgary, Alberta Canada; 4 Department of Pharmacology and Therapeutics, McGill University, Montreal, Quebec Canada; 5 Department of Microbiology and Immunology, McGill University, Montreal, Quebec Canada; 6 Department of Genetics, CHU Sainte-Justine Research Center, Montreal Quebec, Canada; 7 Department of Pathology, McGill University, Montreal, Quebec Canada; 8 McGill International TB Centre, McGill University, Montreal, Quebec Canada

**Keywords:** Alveolar macrophages, Viral infection

## Abstract

Resident-tissue macrophages (RTMs) arise from embryonic precursors^1,2^, yet the developmental signals that shape their longevity remain largely unknown. Here we demonstrate in mice genetically deficient in 12-lipoxygenase and 15-lipoxygenase (*Alox15*^*−/−*^ mice) that neonatal neutrophil-derived 12-HETE is required for self-renewal and maintenance of alveolar macrophages (AMs) during lung development. Although the seeding and differentiation of AM progenitors remained intact, the absence of 12-HETE led to a significant reduction in AMs in adult lungs and enhanced senescence owing to increased prostaglandin E_2_ production. A compromised AM compartment resulted in increased susceptibility to acute lung injury induced by lipopolysaccharide and to pulmonary infections with influenza A virus or SARS-CoV-2. Our results highlight the complexity of prenatal RTM programming and reveal their dependency on in *trans* eicosanoid production by neutrophils for lifelong self-renewal.

## Abstract (web summary)

The production of eicosanoids by neutrophils during the neonatal period is necessary for the self-renewal and maintenance of alveolar macrophages during lung development.

## Main

Alveolar macrophages (AMs) are the major embryonically derived population that reside in the airways and play an essential part in the maintenance of pulmonary homeostasis and immunosurveillance^1–3^. In contrast to interstitial macrophages derived from bone marrow (BM)^4–7^, AMs originate from fetal liver monocytes that seed the lung during embryogenesis and mature during the first week of life^2,8,9^. At steady state, AMs are maintained by self-renewal, with limited contribution from circulating monocytes^2,10^. The essential role of the lung microenvironment, including granulocyte–macrophage colony-stimulating factor (GM-CSF) and transforming growth factor-β (TGFβ) signalling, in AM development and function has been extensively studied^2,9,11^. However, the potential of other BM-derived immune cells and their specific function in the early AM programming is incompletely understood.

Eicosanoids are evolutionarily conserved bioactive lipids. They are essential mediators of homeostatic and inflammatory processes^12^ and potent regulators of macrophage function^13–17^. Eicosanoids are generated through two main pathways: the cyclooxygenase pathway (involving cyclooxygenase 1 (COX1) and COX2) and the lipoxygenase pathway (involving 5-lipoxygenase (ALOX5) and 12-lipoxygenase and 15-lipoxygenase (ALOX12/15)). ALOX15 generates 12-hydroxyeicosatetraenoic acid (12-HETE), 15-HETE and specialized pro-resolving mediators such as lipoxin A_4_. Meanwhile, the cyclooxygenase pathway produces prostaglandins, including prostaglandin E_2_ (PGE_2_)^12^. Although eicosanoid metabolites are produced in the lung at homeostasis^18^ and control macrophage-mediated host defence against pathogens^13,19,20^, little is known about their contribution to lung alveolarization. In the current study, we demonstrate the crucial role of the ALOX15 pathway in neutrophil-dependent AM programming in the developing lung.

## ALOX15 is required for AM maintenance

**Fig. 1:**
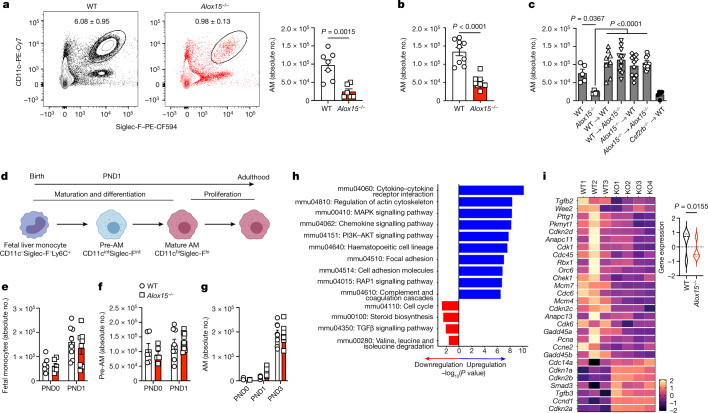
ALOX15 is required for AM maintenance. **a**, Representative FACS plots (left) and quantification (right) of AM numbers (gated on single live cells, CD45.2^+^CD11c^+^Siglec-F^+^) in the lungs of adult WT (*n* = 7) and *Alox15*^*−/−*^ (*n* = 6) mice. **b**, AM numbers in BAL (*n* = 9 mice per group). **c**, Quantification of the AM population in the lungs of BM chimeras 8 weeks after reconstitution (left to right, *n* = 5, 5, 8, 11, 11, 9 and 5 mice per group, respectively). **d**, Model of AM development. **e**–**g**, Numbers of fetal monocytes (**e**), pre-AMs (**f**) and AMs (**g**) in WT and *Alox15*^*−/−*^ lungs at various ages (*n* = 6 (PND0) or 8 (PND1 and PND3) per group). **h**, Top upregulated and downregulated pathways from KEGG Pathway enrichment analysis. **i**, Violin plot (left) and heatmap (right) of mRNA transcripts significantly upregulated and downregulated in the cell cycle pathway (*n* = 3 (WT) or 4 (*Alox15*^*−/−*^)). Data are presented as the mean ± s.e.m. and are from one experiment (**h**,**i**) or pooled from two (**a**,**c**,**e**–**g**) or three (**b**) independent experiments. Data were analysed using unpaired two-tailed *t*-test (**a**,**b**), Mann–Whitney two-tailed test (**i**) or one-way analysis of variance (ANOVA) followed by Tukey’s multiple comparisons test (**c**). The model in **d** was created using BioRender (https://biorender.com). Source data

**Extended Data Fig. 1:**
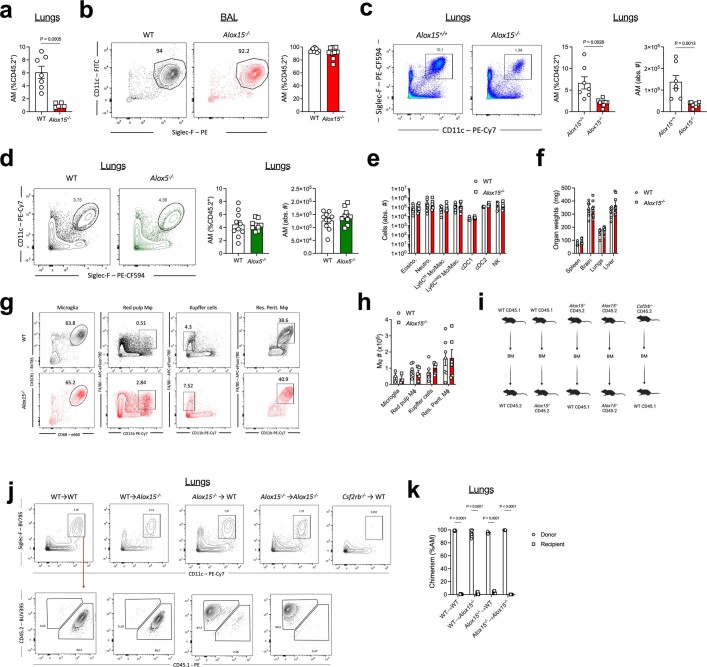
ALOX15, but not ALOX5, is required for AM homeostasis. (a) AM frequencies in the lungs of adult WT (n = 7) and *Alox15*^*−/−*^ (n = 6) mice. (b) Representative FACS plot and quantification of AM frequencies in the BAL of adult WT and *Alox15*^*−/−*^ mice (n = 9 mice/group). (c) Representative FACS plot and quantification of AM frequencies and numbers in the lungs of adult *Alox15*^*−/−*^ (n = 9) and *Alox15*^*+/+*^ (n = 7) littermate control mice. (d) Representative FACS plot and quantification of AM frequencies and numbers in the lungs of adult WT (n = 10) and *Alox5*^*−/−*^ (n = 9) mice. (e) Quantification of lung innate cells in adult WT and *Alox15*^*−/−*^ mice (n = 8/8/8/8/6/6/6/group). See also Supplementary Fig. 1 for gating strategy. (f) Weight of spleen, lungs, brain and top liver lobe from WT and *Alox15*^*−/−*^ adult mice (n = 8 mice/group). (g-h) Representative FACS plot (g) and quantification (h) of resident tissue macrophages in adult WT (n = 3/6/7/7/group) and *Alox15*^*−/−*^ mice (n = 3/5/6/6/group). (i) Bone marrow chimera model. (j-k) Representative FACS plots (j) and AM chimerism 8 weeks post-BM reconstitution (right) (n = 8/11/11/9/group). Data are presented as mean ± s.e.m and are pooled from two (a, c-e, f-k) or three (b-e) independent experiments and were analyzed using unpaired two-tailed t-test (a, c) or two-way ANOVA followed by Sidak’s multiple comparisons test (k). The model in (i) was created using BioRender (https://biorender.com). Source data

The frequency and number of AMs in *Alox15*^*−/−*^ adult mice (6–8 weeks old) were significantly decreased (by about 50%) compared with wild-type (WT) controls at steady state (Fig. 1a,b and Extended Data Fig. 1a–c). This reduction was specific to the ALOX15 pathway, as the AM compartment was intact in ALOX5-deficient (*Alox5*^*−/−*^) mice (Extended Data Fig. 1d). Notably, the effect of ALOX15 deficiency was specific to AMs, as there was no difference in other populations of lung innate immune cells (Extended Data Fig. 1e and Supplementary Fig. 1) and extrapulmonary RTMs (Extended Data Fig. 1f–h). To validate this observation, we generated BM chimeras in which AMs are replenished by BM-derived monocytes^2,10^. Lethally irradiated WT (CD45.1 or CD45.2) and *Alox15*^*−/−*^ (CD45.2) mice were reconstituted with BM from *Alox15*^*−/−*^, *Csf2rb*^*−/−*^ or WT CD45.1 donor mice (Extended Data Fig. 1i). Following 8 weeks of BM reconstitution, AMs were replenished by donor cells, and there was no difference in AM development between groups. The exception was in mice reconstituted by *Csf2rb*^*−/−*^ BM, which were used as a control^2,21^ (Fig. 1c and Extended Data Fig. 1j–k). Thus, the reduction in AMs in *Alox15*^*−/−*^ mice is linked to embryonic development.

## Impaired proliferation in *Alox15*^*−/−*^ AMs

**Extended Data Fig. 2:**
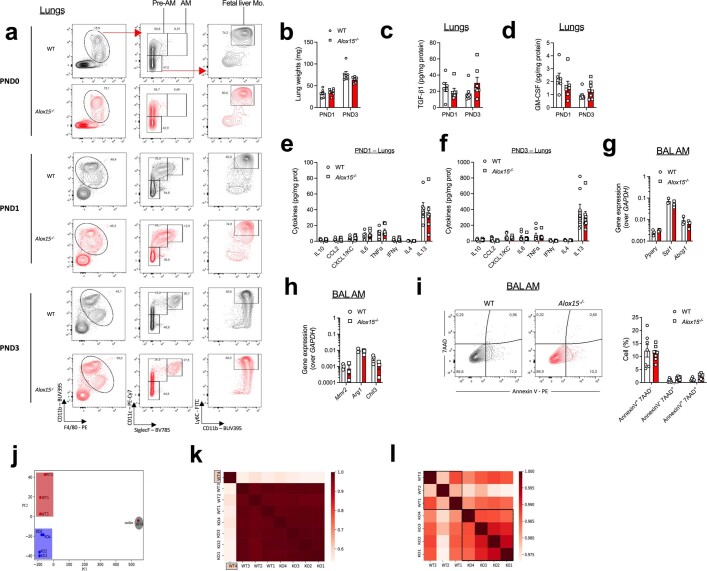
*Alox15*^*−/−*^ AM seeding and maturation. (a) Representative FACS plot for the determination of AM progenitors (pre-AM and fetal liver monocytes) and AM in the lungs of PND0, PND1 and PND3 WT and *Alox15*^*−/−*^ mice. (b) Weight of lungs from PND1 and PND3 WT (n = 7/group) and *Alox15*^*−/−*^ mice (n = 6/group). (c-f) Lungs from PND1 or PND3 WT and *Alox15*^*−/−*^ mice were harvested and the levels of TGF-β1 (c) (PND1, WT: n = 6, *Alox15*^*−/−*^: n = 7; PND3, WT: n = 8, *Alox15*^*−/−*^: n = 7), GM-CSF (d) (PND1, WT: n = 6, *Alox15*^*−/−*^: n = 7; PND3, WT: n = 8, *Alox15*^*−/−*^: n = 8), type 1 and type 2 cytokines (e- PND1: WT: n = 6, *Alox15*^*−/−*^: n = 7, f- PND3: n = 8/group) were determined by ELISA. (g-h) BAL-AM were isolated from PND3 WT and *Alox15*^*−/−*^ mice for gene expression analysis (n = 3 biological replicates/group). (i) BAL-AM were isolated from adult naïve animals and cell death was assessed by flow cytometry using AnnexinV/7AAD staining (n = 7/group). (j-l) Batch effect examinations. (j) PCA and (k) heatmap plots that suggest WT4 is an outlier. (l) A heatmap that indicates the similarity between different samples. Data are presented as mean ± s.e.m and are pooled from two (b-f, i) or three (g-h) independent experiments. Source data

Because AMs develop from fetal liver monocytes during the first days of life^2^ (Fig. 1d), we analysed the progenitors (fetal liver monocytes, pre-AMs) and mature AMs of WT and *Alox15*^*−/−*^ mice at postnatal day 0 (PND0), PND1 and PND3 (Fig. 1e–g and Extended Data Fig. 2a). There was no difference in the number of progenitors or mature AMs between WT and *Alox15*^*−/−*^ mice (Fig. 1e–g). There was also no difference in pulmonary levels of the key AM maturation cytokines GM-CSF and TGFβ1 as well as other cytokines (Extended Data Fig. 2b–f) at PND1 and PND3. Moreover, the expression of key AM genes (*Abcg1*, *Pparg*, *Spi1*, *Mmr*, *Ym1* and *Chil3*) (Extended Data Fig. 2g,h) were comparable in PND3 AMs between WT and *Alox15*^*−/−*^ mice.

**Fig. 2:**
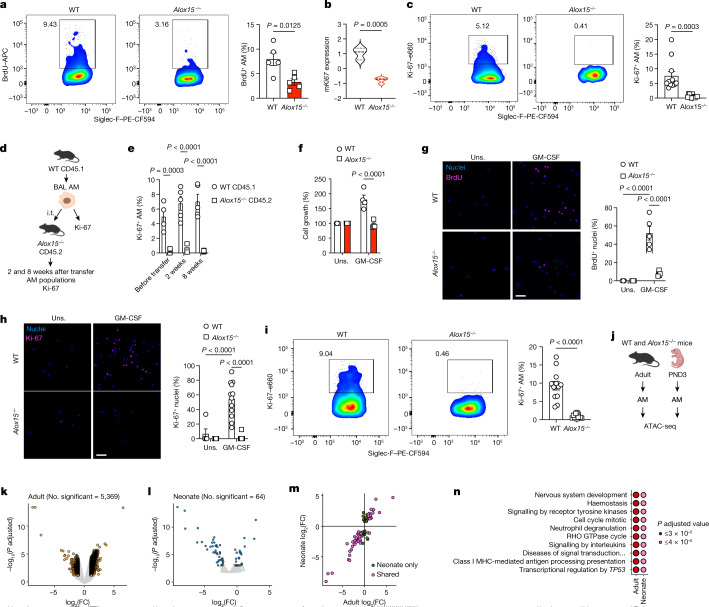
*Alox15*^*−/−*^ AMs are intrinsically impaired in proliferation. **a**, Representative FACS plots (left) and quantification (right) of BrdU^+^ AMs in adult WT and *Alox15*^*−/−*^ lungs after a 7-day BrdU pulse (*n* = 5 mice per group). **b**, Mouse *Ki67* (*mKi67*) expression from the RNA-seq dataset (*n* = 3 (WT) or 4 (*Alox15*^*−/−*^)). **c**, Representative FACS plots (left) and quantification (right) of Ki-67^+^ AMs in adult WT and *Alox15*^*−/−*^ lungs (*n* = 11 per group). **d**, Scheme of the adoptive transfer protocol. **e**, Ki-67^+^ AMs before and after transfer (WT CD45.1 (left to right), *n* = 4, 5 or 5; *Alox15*^*−/−*^ CD45.2, *n* = 5 per group). **f**, Growth of AMs after culture with GM-CSF for 3 days (*n* = 4 biological replicates per group). **g**,**h**, Representative micrographs (left) and quantification (right) of BrdU^+^ (**g**) (*n* = 3 (unstimulated (Uns.) or 5 (GM-CSF) fields of view) and Ki-67^+^ (**h**) (*n* = 5 (Uns.), 12 (WT GM-CSF) or 10 (*Alox15*^*−/−*^ GM-CSF) fields of view) AMs after 3 days of culture with GM-CSF. Scale bar, 50 µm. **i**, Representative FACS plots (left) and quantification (right) of Ki-67^+^ AMs in PND3 lungs (*n* = 12 mice per group). **j**, Scheme of ATAC-seq of AMs from adult (BAL) or PND3 (sorted) WT and *Alox15*^*−/−*^ mice (*n* = 3 mice per group). **k**,**l**, Volcano plots for differential accessibility results in adult (**k**; knockout (KO) versus WT) and pups (**l**). Yellow (**k**) or blue (**l**) highlighted peaks have a *P*-adjusted value of <0.05 and absolute log_2_(fold change (FC)) of ≥1. **m**, The log_2_(FC) difference in accessibility in adults and pups for all significant peaks within pups. Pink highlighted points are DA in both adults and pups. Green points are significant only in pups. **n**, Top ten enriched pathways in adults and pups from a GSEA of genes matched to the closest DA peaks. Data are presented as the mean ± s.e.m. and are from one (**e**,**k**–**n**) or pooled from three (**c**,**i**) or four (**f**) independent experiments or representative of two (**a**) or three (**g**,**h**) independent experiments. Data were analysed using unpaired two-tailed *t*-test (**a**–**b**,**i**) or two-way ANOVA followed by Sidak’s (**e**) or Tukey’s (**f–h**) multiple comparisons test. The models in **d** and **j** were created using BioRender (https://biorender.com). Source data

**Extended Data Fig. 3:**
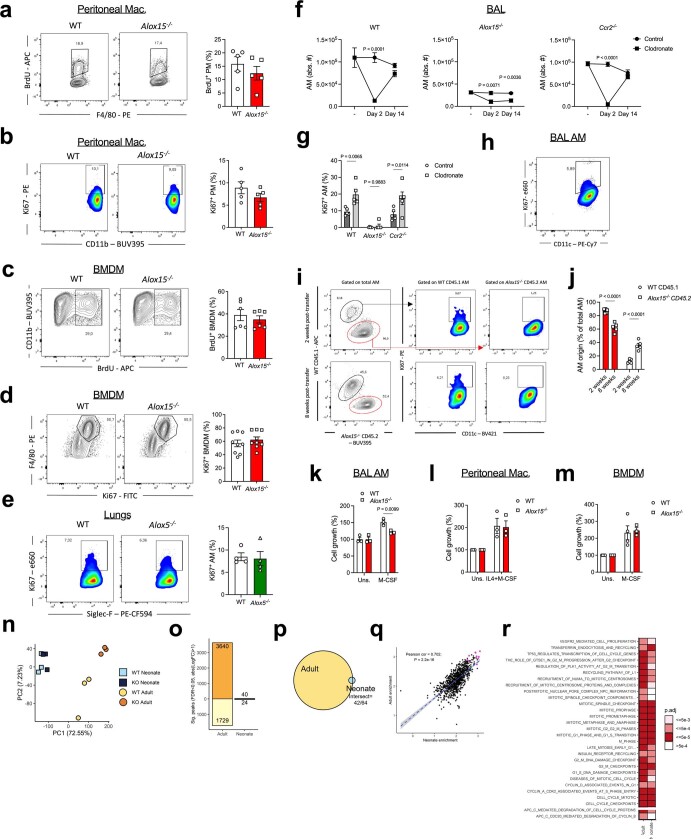
Intact PM and BMDM proliferation. (a-b) Peritoneal macrophages (PM) were harvested from adult naïve WT and *Alox15*^*−/−*^ mice and BrdU incorporation after 7 days pulse (a) or Ki67 expression (b) were determined by flow cytometry (n = 5/group). (c-d) Bone marrow-derived macrophages were generated from adult WT and *Alox15*^*−/−*^ mice and were treated with BrdU (day 3 and 5 of differentiation). At day 6 BrdU incorporation (c) (n = 6/group) and Ki67 expression (d) (n = 9/group) were evaluated by flow cytometry. (e) Representative FACS plot and quantification of Ki67^+^ AM frequencies in the lungs of adult WT and *Alox5*^*−/−*^ mice (n = 4/group). (f-g) AM kinetics following local depletion by clodronate liposomes. Adult WT (control: n = 3/6/9/timepoint, clodronate: n = 3/6/10/timepoint), *Alox15*^*−/−*^ (control: n = 3/6/10 mice/timepoint, clodronate: n = 3/6/9/timepoint) or *Ccr2*^*−/−*^ (n = 3/3/5/group/timepoint) mice were given control or clodronate liposomes intranasally (70 µl). AM populations were assessed in the airways at day 2 and 14 post-delivery. (f) Numbers of AM in the BAL at various days post-infection. (g) Frequency of Ki67^+^ AM in the BAL at day 14 (n = 5/group). (h) Representative FACS plot of Ki67^+^ CD45.1 BAL AM before adoptive transfer. (i) Representative FACS plots of AM in the lungs of *Alox15*^*−/−*^ mice 2 and 8 weeks after WT CD45.1 AM transfer. (j) Quantification of WT CD45.1^+^ or *Alox15*^*−/−*^ CD45.2^+^ AM post-adoptive transfer in ALOX15-deficient mice (n = 5/group). (k) BAL AM from adult WT and *Alox15*^*−/−*^ mice were cultured with M-CSF for 3 days before cell growth was evaluated (n = 3 biological replicates/group). (l) PM isolated from adult WT and *Alox15*^*−/−*^ mice were cultured with M-CSF+IL-4 for 3 days before cell growth was evaluated (n = 3 biological replicates/group). (m) BMDM generated from adult WT (n = 4 biological replicates) and *Alox15*^*−/−*^ (n = 3 biological replicates) mice were cultured with M-CSF for 3 days before cell growth was evaluated. (n-r) WT and *Alox15*^*−/−*^ AM from adult and PND3 mice were subjected to ATAC-Seq (n = 3/group). (n) Principal component analysis of WT versus KO adult and pup cells. (o) The number of differentially accessible peaks comparing ALOX15-deficient and WT cells using the cutoffs: p.adj < 0.05 and abs(log2FC) > 1. Upward bars indicate increased accessibility in the KO condition and downward bars indicate decreased accessibility. (p) Venn diagram showing the relative numbers of DA peaks in adults and pups. Circle size is proportional to the number of DA peaks. Out of 64 total peaks with p.adj < 0.05 and abs(log2FC) > 1 in pups, ~65% (42/64) overlapped with the DA peaks set in adults. (q) Correlation (pearson cor = 0.702, p < 2.2e-16) of normalized enrichment scores (NES) for all enriched pathways in adults and pups. Pink highlighted points are the top 10 pathways in both adults and pups. (r) Heatmap showing the gene set enrichment results specifically for reactome pathways related to proliferation. Shading is indicative of significance with darker red colors indicating lower p.adjusted values. Data are presented as mean ± s.e.m and are from one (e, g-j, n-r), or pooled from two (c, f) or three (d, k-m) or representative of two (a-b) independent experiments. Data were analyzed using two-way ANOVA followed by Tukey’s (f) or Sidak’s (g, j-k) multiple comparisons test. Source data

As the loss of AMs in adult ALOX15-deficient mice was independent of AM seeding, maturation or differentiation and aberrant cell death (Extended Data Fig. 2i), we next performed bulk RNA sequencing (RNA-seq) to assess the overall effect of ALOX15 deficiency in AMs. We identified 503 differentially expressed genes (false discovery rate (FDR) adjusted *P* value < 0.05), which included 346 upregulated genes (between WT and *Alox15*^*−/−*^ mice) and 157 downregulated genes. Pathway enrichment analysis revealed a significant (*P* = 0.0021) downregulation of cell cycle genes in *Alox15*^*−/−*^ AMs (Fig. 1h,i, Extended Data Fig. 2j–l and Supplementary Table 1). This transcriptomics analysis was further supported by a significant reduction in BrdU incorporation and undetectable Ki-67 in AMs isolated from *Alox15*^*−/−*^ mice (Fig. 2a–c). This was specific to *Alox15*^*−/−*^ AMs, as no differences in proliferation were observed in *Alox15*^*−/−*^ peritoneal macrophages (PMs) or BM-derived macrophages (BMDMs) (Extended Data Fig. 3a–d). Notably, ALOX5 deficiency had no effect on the frequency of Ki-67^+^ AMs (Extended Data Fig. 3e). Finally, we locally depleted AMs using clodronate liposomes (administered intranasally) and followed the dynamics of the AM populations at day 2 (peak of depletion) and day 14 to evaluate their proliferation capacity for repopulating the alveolar space. AMs were depleted at day 2 after clodronate delivery in all groups. By contrast, by day 14, AM numbers from WT and *Ccr2*^*−/−*^ mice returned to levels that were similar to that of control mice (Extended Data Fig. 3f). This result indicated that AMs are able to proliferate (Extended Data Fig. 3g) and repopulate the airways independent of BM monocytes^10^. However, *Alox15*^*−/−*^ AMs were not able to proliferate or repopulate the airways (Extended Data Fig. 3f,g). Thus, during homeostasis or local depletion, the self-renewing capacity of AMs is impaired in ALOX15-deficient mice.

The impaired proliferative capacity of *Alox15*^*−/−*^ AMs can be due to either extrinsic signalling (that is, altered eicosanoid composition of the pulmonary microenvironment) or intrinsic signalling. However, the pulmonary microenvironment of *Alox15*^*−/−*^ mice did not suppress the proliferation of AMs. That is, adoptive transfer of WT CD45.1 AMs into *Alox15*^*−/−*^ mice (Fig. 2d) repopulated the ‘incomplete’ *Alox15*^*−/−*^ AM niche and remained proliferative (Fig. 2e and Extended Data Fig. 3h–j). To examine the intrinsic capacity of AMs to proliferate, we first cultured WT and *Alox15*^*−/−*^ AMs with GM-CSF or M-CSF and found that *Alox15*^*−/−*^ AMs remained non-proliferative (Fig. 2f–h and Extended Data Fig. 3k). This phenotype was specific to AMs, as the proliferation of *Alox15*^*−/−*^ PMs and BMDMs was intact (Extended Data Fig. 3l–m). In vivo, the impaired proliferation in AMs was detected as early as PND3, the development stage at which mature AMs first appear in the airways (Fig. 2i). Next, we generated assay for transposase-accessible chromatin with high-throughput sequencing (ATAC-seq) data from neonatal (PND3) and adult AMs from both WT and *Alox15*^*−/−*^ mice (Fig. 2j and Extended Data Fig. 3n). ALOX15 deficiency altered the chromatin accessibility landscape of AMs in both neonatal and adult mice. Moreover, the smaller numbers of chromatin changes identified in neonates were amplified in adult AMs. In adult AMs, significant differential chromatin accessibility was detected at 5,369 sites (Fig. 2k and Extended Data Fig. 3o; adjusted *P* < 0.05, absolute(log_2_(fold change)) > 1). This result demonstrates that *Alox15* plays an overall crucial role in modulating the epigenetic landscape of AMs. Notably, although we detected fewer differentially accessible (DA) sites in neonatal *Alox15*^*−/−*^ AMs (Fig. 2l and Extended Data Fig. 3o), 42 out of 64 of those sites overlapped DA sites in the adult AMs (Extended Data Fig. 3p). This result represented a more than sevenfold increase over what one would expect by chance (*P* < 1 × 10^−10^). Moreover, effect sizes and the directionality of this core set of shared DA peaks were highly correlated when comparing adult with neonate AMs (Fig. 2m). Gene set enrichment analysis (GSEA) performed on the genes closest to DA peaks demonstrated that the top ten most enriched pathways were identical when comparing adult with neonate AMs (Fig. 2n and Extended Data Fig. 3q). The pathway ‘cell cycle mitotic’ was among these top pathways, which was in agreement with our functional data showing that ALOX15 deficiency alters cell proliferation. Moreover, many other pathways directly related to cell division were significantly enriched in both neonate and adult AMs (Extended Data Fig. 3r). Collectively, these data support the notion that ALOX15 signalling imprints epigenetic changes in neonate AMs that are retained and strengthened during development to adult AMs.

## *Alox15*^*−/−*^ AMs are senescent

**Extended Data Fig. 4:**
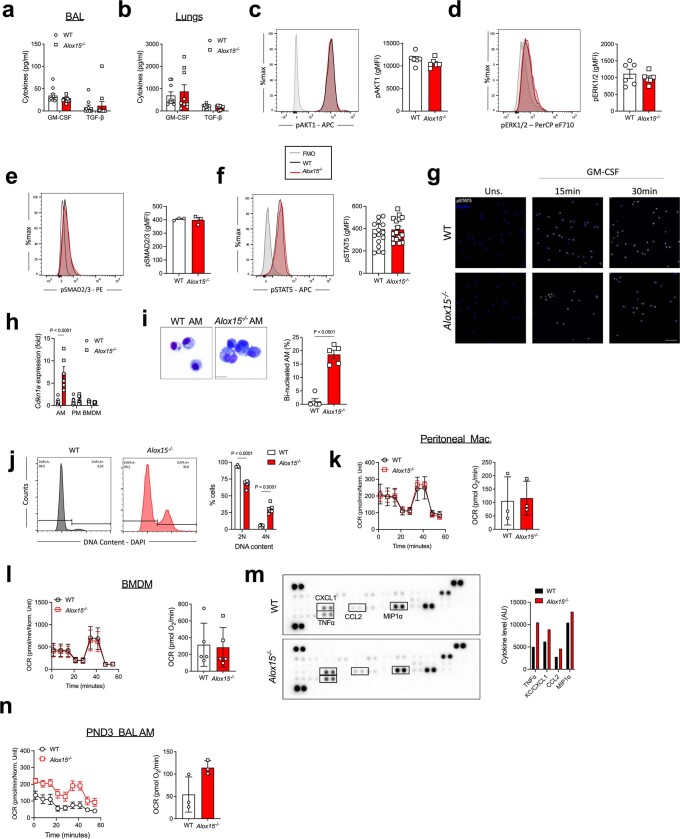
Intact GM-CSF and TGF-β1 signalling in *Alox15*^*−/−*^ AM. BAL (a) (WT: n = 11/10/group, *Alox15*^*−/−*^: n = 12/11/group) and lung tissue (b) (WT: n = 9/group, *Alox15*^*−/−*^: n = 9/8/group) levels of GM-CSF and TGF-β1 in adult WT and *Alox15*^*−/−*^ mice. (c-f) BAL-AM were isolated from adult naïve animals and phosphorylation of pAKT1 (c) (n = 6/group), pERK1/2 (d) (n = 6/group), pSMAD2/3 (e) (n = 3/group), pSTAT5 (f) (WT: n = 17, *Alox15*^*−/−*^: n = 16) were assessed by flow cytometry. (g) BAL-AM were isolated from adult naïve animals and cultured with GM-CSF (10 ng/ml) for various time points before phosphorylation of STAT5 was evaluated. Bar = 50 µm. (h) Expression of *Cdkn1a* in AM, PM and BMDM from adult WT and *Alox15*^*−/−*^ mice by qPCR (n = 6/group). (i) Left, representative micrographs of H&E-stained adult WT and *Alox15*^*−/−*^ AM harvested by BAL. Bar = 10 µm. Right, percentage of bi-nucleated cells in BAL AM (n = 5 fields of view/group). (j) Representative histograms (left) and quantification (right) of DNA content in BAL AM from adult WT and *Alox15*^*−/−*^ mice (n = 6/group). (k) Representative curve (left, n = 3/group) and quantification (right, n = 3 biological replicates/group) of OCR in PM isolated from adult WT and *Alox15*^*−/−*^ mice. (l) Representative curve (left, n = 5/group) and quantification (right, n = 5 biological replicates/group) of OCR in BMDM generated from adult WT and *Alox15*^*−/−*^ mice. (m) Representative immunoblot (left) and quantification (right) of cytokine production in the supernatant of resting (24 h) pooled from adult WT or *Alox15*^*−/−*^ BAL AM. (n) Representative curve (left, WT: n = 5, *Alox15*^*−/−*^: n = 3) and quantification (right, n = 3 biological replicates/group) of OCR in AM from PND3 WT and *Alox15*^*−/−*^ mice. Data are presented as mean ± s.e.m and are from one (e, m) or pooled from two (c-d, h, j), three (a-b, k-l, n), or five (f, i) independent experiments or representative of two (g) independent experiments. Data were analyzed using unpaired two-tailed t-test (i) or two-way ANOVA followed by Sidak’s multiple comparisons test (h, j). Source data

Pathways involved in GM-CSF and TGFβ signalling are key players in the proliferation and differentiation of AMs^9,11^. However, GM-CSF and TGFβ1 production and phosphorylation of AKT1, ERK1, ERK2, SMAD2 and SMAD3 were unchanged between *Alox15*^*−/−*^ and WT AMs (Extended Data Fig. 4a–e). Furthermore, WT and *Alox15*^*−/−*^ AMs had similar STAT5 phosphorylation levels at steady state (Extended Data Fig. 4f) and after GM-CSF stimulation (Extended Data Fig. 4g). Thus, impaired proliferation of *Alox15*^*−/−*^ AMs occurs independent of GM-CSF or TGFβ signalling.

**Fig. 3:**
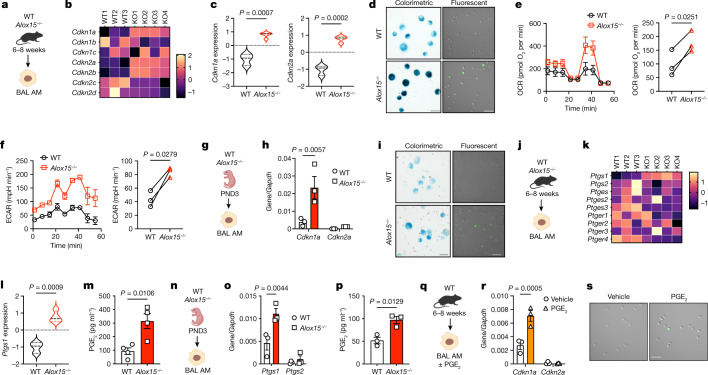
*Alox15*^*−/−*^ AMs are senescent. **a**, Scheme of BAL-isolated AMs from adult (6–8 weeks) WT and *Alox15*^*−/−*^ mice. **b**,**c**, Heatmap of selected mRNA transcripts of cell cycle inhibitors (**b**) and expression of *Cdkn1a* and *Cdkn2a* (**c**) in adult WT and *Alox15*^*−/−*^ AMs (*n* = 3 (WT) or 4 (*Alox15*^*−/−*^)). **d**, Representative micrographs of SA-β-galactosidase expression assessed by colorimetric or fluorescence assays. Scale bars, 25 µm (left) and 50 µm (right). **e**,**f**, Representative curves (left) and quantification (right) of the oxygen consumption rate (OCR) (**e**) and the extracellular acidification rate (ECAR) (**f**) (*n* = 3 biological replicates/group). **g**–**i**, Scheme of experiment (**g**), *Cdkn1a* and *Cdkn2a* expression (**h**) (*n* = 3 biological replicates per group) and SA-β-galactosidase expression (**i**) in AMs from PND3 WT and *Alox15*^*−/−*^ mice. Scale bars, 25 µm (left) and 50 µm (right). **j**–**l**, Scheme of experiment (**j**), heatmap of selected mRNA transcripts of the prostanoid pathway (**k**) and expression of *Ptgs1* (**l**) from adult WT and *Alox15*^*−/−*^ AMs (*n* = 3 (WT) or 4 (*Alox15*^*−/−*^)) in bulk RNA-seq. **m**, PGE_2_ production by resting AMs (24 h) from WT and *Alox15*^*−/−*^ mice (*n* = 4 biological replicates per group). **n**,**o**, Scheme of experiment (**n**) and *Ptgs1* and *Ptgs2* expression (**o**) by AMs from WT and *Alox15*^*−/−*^ PND3 mice (*n* = 3 biological replicates per group). **p**, PGE_2_ production by resting AMs (24 h) from WT and *Alox15*^*−/−*^ PND3 mice (*n* = 3 biological replicates per group). **q**, BAL AMs were isolated from adult (6–8 weeks) WT mice, and the AMs were cultured with or without GM-CSF (20 ng ml^–1^) for 3 days and treated or with or without 10 µM of exogenous PGE_2_. **r**,**s**, *Cdkn1a* and *Cdkn2a* expression (**e**) (*n* = 3 biological replicates per group) and SA-β-galactosidase expression (**s**). Scale bar, 50 µm. Data are presented as the mean ± s.e.m. and are from one experiment (**b**,**c**,**k**–**l**) or from three (**e**,**f**,**h**,**o**,**p**,**r**) or four (**m**) independent experiments, or representative of two (**d**,**l**,**s**) independent experiments. Data were analysed using unpaired (**c**,**l**,**m**,**p**) or paired (**e**,**f**) two-tailed *t*-test or two-way ANOVA followed by Sidak’s multiple comparisons test (**h**,**o**,**r**). The models in **a**, **g**, **j** and **n** were created using BioRender (https://biorender.com). Source data

Various biological processes can lead to permanent cell cycle arrest, including cellular senescence^22^. Bulk RNA-seq analysis of adult WT and *Alox15*^*−/−*^ AMs showed that the expression of two key cell cycle inhibitors, *Cdkn1a* and *Cdkn2a*, was significantly increased in adult *Alox15*^*−/−*^ AMs (Fig. 3a–c). This was specific to AMs, as there was no difference in *Cdkn1a* expression levels between WT and *Alox15*^*−/−*^ PMs or BMDMs (Extended Data Fig. 4h). In support of cellular senescence, the expression of senescence-associated β-galactosidase (SA-β-galactosidase) and the frequency of binucleated cells were also significantly increased in *Alox15*^*−/−*^ AMs (Fig. 3d and Extended Data Fig. 4i–j). Cellular senescence is often associated with metabolic reprogramming and increased cytokine production^23^. Both basal mitochondrial respiration and glycolysis were increased in *Alox15*^*−/−*^ AMs (Fig. 3e,f), but not *Alox15*^*−/−*^ PMs or BMDMs (Extended Data Fig. 4k–l). This increased metabolic activity at steady state was associated with increased production of pro-inflammatory cytokines in *Alox15*^*−/−*^ AMs (Extended Data Fig. 4m). Furthermore, the senescence of *Alox15*^*−/−*^ AMs initiated during early development, as PND3 *Alox15*^*−/−*^ AMs showed increased *Cdkn1a* and SA-β-galactosidase expression and cellular metabolism (Fig. 3g–i and Extended Data Fig. 4n) but reduced proliferation (Fig. 2i). Thus, during lung development, ALOX15-deficient AMs become senescent and undergo irreversible cell cycle arrest.

## PGE_2_ induces senescence in AMs

**Extended Data Fig. 5:**
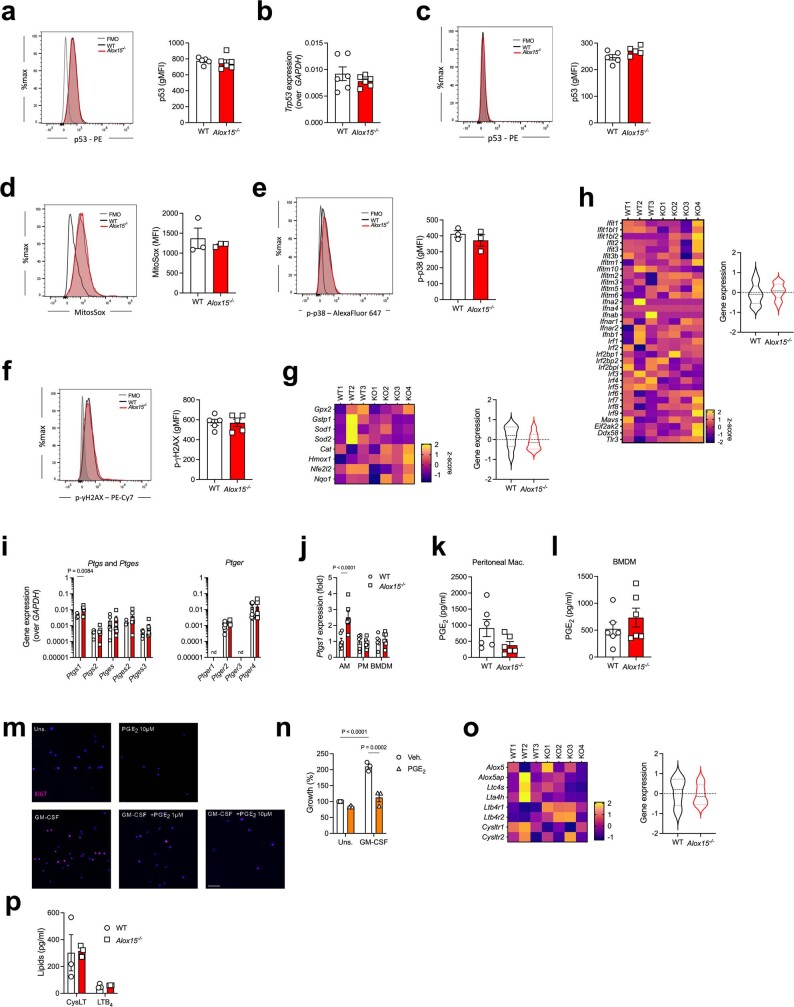
No difference in canonical senescence pathways. (a) Representative histograms and quantification of p53 MFI in PND3 AM from WT (n = 5) and *Alox15*^*−/−*^ (n = 6) animals. (b) Expression of *Trp53* in adult AM from WT and *Alox15*^*−/−*^ mice (n = 6/group). (c-f) Representative histograms and quantification of p53 (c) (n = 5/group), MitoSox (d) (n = 3/group), p-p38 (e) (n = 3/group) and p-γH2AX (f) (n = 5/group) in BAL AM isolated from adult WT and *Alox15*^*−/−*^ mice. (g-h) Heatmap and quantification of genes involved in antioxidant response (g) or interferon response (h) in BAL AM isolated from adult WT (n = 3) and *Alox15*^*−/−*^ (n = 4) mice in bulk RNA-Seq dataset. (i) Expression of *Ptgs*/*Ptges* (WT n = 5/group, *Alox15*^*−/−*^ n = 6/5/6/6/6/group) and *Ptger* genes by BAL AM from adult WT and *Alox15*^*−/−*^ mice (n = 6/group). (j) Expression of *Ptgs1* by AM, PM and BMDM from adult WT and *Alox15*^*−/−*^ mice (n = 6/group). (k-l) PGE_2_ production by naïve resting (24 h) peritoneal macrophages (k) or bone marrow derived macrophages (l) from adult WT and *Alox15*^*−/−*^ mice (n = 6/group). (m-n) BAL AM were isolated from adult (6–8 weeks) WT mice, and they were cultured or not with GM-CSF (20 ng/ml) for 3 days and treated or not with various concentrations of exogenous PGE_2_. (m) Representative micrographs of Ki67^+^ AM after 3 days of culture with GM-CSF and treated or not with PGE_2_ (1–10 µM). Bar = 50 µm. (n) AM growth was assessed after culture with GM-CSF and treatment with PGE_2_ (10 µM) (n = 3 biological replicates/group). (o) Heatmap and quantification of lipoxygenase pathway related genes in BAL AM from adult WT (n = 3) and *Alox15*^*−/−*^ (n =  4). (p) Production of LTB_4_ and cysteinyl leukotrienes by resting adult WT and *Alox15*^*−/−*^ BAL AM (24 h) (n = 3 biological replicates/group). Data are presented as mean ± s.e.m and are from one (e, g-h, o) or pooled from two (b, i-l) or three (n, p) independent experiments or representative of two experiments (a, c-f, m) independent experiments. Data were analysed using two-way ANOVA followed by Sidak’s multiple comparisons test (i-j, n). Source data

Cellular senescence can be triggered by oxidative stress or a DNA damage response, which leads to increased p53 expression. However, there was no difference in the expression or protein levels of p53 in PND3 (Extended Data Fig. 5a) or adult (Extended Data Fig. 5b,c) WT and *Alox15*^*−/−*^ AMs. This was consistent with similar levels of mitochondrial reactive oxygen species, p38 phosphorylation, the DNA damage marker phospho-γ-H2AX and the oxidative stress response (Extended Data Fig. 5d–g). In addition, there was no difference in the expression of type I interferons (IFN-I) or interferon-stimulated genes (Extended Data Fig. 5h), which has been recently shown to promote senescence^24,25^.

Increased PGE_2_ production^26^ has been shown to suppress the proliferation of AMs^27^ and to maintain the cellular senescence state^28^. Consistently, *Ptgs1* (which encodes COX1) expression and production of PGE_2_ were significantly increased in PND3 and adult *Alox15*^*−/−*^ AMs, but not adult *Alox15*^*−/−*^ PMs or BMDMs (Fig. 3j–p and Extended Data Fig. 5i–l). PGE_2_ production was responsible for inducing the senescence of AMs, as treatment of WT AMs (Fig. 3q) with exogenous PGE_2_ substantially decreased GM-CSF-induced Ki-67 expression (Extended Data Fig. 5m), suppressed GM-CSF-mediated proliferation of AMs (Extended Data Fig. 5n) and increased the expression of *Cdkn1a* and SA-β-galactosidase (Fig. 3r,s). Cellular senescence of *Alox15*^*−/−*^ AMs was specific to PGE_2_, as expression of the lipoxygenase pathway and its mediators (leukotriene B_4_ (LTB_4_) and cysteinyl leukotrienes) remained unchanged (Extended Data Fig. 5o,p). Thus, an increase in the production of PGE_2_ induces senescence in *Alox15*^*−/−*^ AMs.

## *Alox15*^*−/−*^ AM function is impaired

**Fig. 4:**
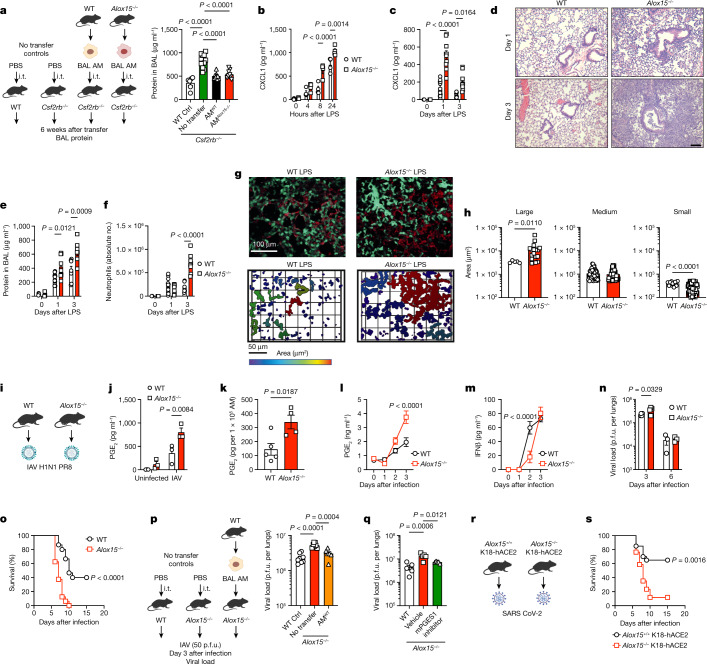
*Alox15*^*−/−*^ AMs have dysregulated responses to sterile inflammation and viral infection. **a**, WT or *Alox15*^*−/−*^ AMs were transferred (intratracheal (i.t.)) into *Csf2rb*^*−/−*^ mice, and BAL protein levels were determined 6 weeks after transfer (left to right, *n* = 6, 11, 9 or 9 per group). **b**, CXCL1 production by WT or *Alox15*^*−/−*^ AMs after stimulation with LPS (100 ng ml^–1^) (*n* = 4 biological replicates per group). **c**–**f**, LPS (20 µg) was delivered intranasally to adult WT and *Alox15*^*−/−*^ mice. **c**, BAL CXCL1 levels (left to right, *n* = 3, 11 or 7 (WT) or 3, 10 or 8 (*Alox15*^*−/−*^) mice per time point). **d**, Pulmonary pathology after LPS treatment. Scale bar, 100 µm. **e**, BAL protein levels (left to right, *n* = 3, 11 or 7 (WT) or 3, 9 or 7 (*Alox15*^*−/−*^) mice per time point). **f**, BAL neutrophil numbers (left to right, *n* = 3, 11 or 7 (WT) or 3, 10 or 8 (*Alox15*^*−/−*^) mice per time point). **g**,**h**, Intravital microscopy following 2 h of intravenous LPS (20 µg) treatment. **g**, Intravascular neutrophil (cyan) aggregation (top) and cluster formation quantified (bottom) by defining contiguous objects and measuring the areas (blue, low; red, high area clusters). Vascular endothelium is red. **h**, Clusters were classified as large (≥3,000 µm^2^) (*n* = 5 (WT) or 17 (*Alox15*^*−/−*^) cells), medium (500–2,999 µm^2^) (*n* = 105 (WT) or 66 (*Alox15*^*−/−*^) cells) or small or individual neutrophils (500 µm^2^) (*n* = 16 (WT) or 161 (*Alox15*^*−/−*^) cells). *n* = 3 per group with 3 fields of view per mouse. **i**,**j**, Scheme (**i**) and quantification (**j**) of PGE_2_ production by WT and *Alox15*^*−/−*^ AMs 4 h after IAV infection (multiplicity of infection of 1) (*n* = 3 biological replicates per group). **k**, PGE_2_ production by BAL AMs from IAV-infected mice (day 1, 50 p.f.u.) after 4 h of culture (*n* = 5 (WT) or 4 (*Alox15*^*−/−*^)). **l**–**n**, WT and *Alox15*^*−/−*^ mice were infected with IAV (50 p.f.u.). **l**, BAL levels of PGE_2_ (left to right, *n* = 6, 9, 9 or 8 (WT) or 5, 7, 9 or 7 (*Alox15*^*−/−*^) per time point). **m**, IFNβ (left to right, *n* = 3, 7, 9 or 8 (WT) or 3, 6, 10 or 7 (*Alox15*^*−/−*^) per time point). **n**, Pulmonary viral loads (*n* = 4 (day 3) or 3 (day 6) mice per group). **o**, Survival of WT (*n* = 15) and *Alox15*^*−/−*^ (*n* = 16) animals infected with IAV (90 p.f.u.). **p**, WT AMs were transferred into *Alox15*^*−/−*^ mice. Animals were infected 2 h after transfer with IAV (50 p.f.u.). Pulmonary viral loads were determined 3 days after infection (left to right, *n* = 8, 7 or 6 per group). **q**, Pulmonary viral loads in IAV-infected (50 p.f.u.) WT and *Alox15*^*−/−*^ mice (treated or with or without mPGES1 inhibitor) (*n* = 5 per group). **r**,**s**, Scheme (**r**) and survival of *Alox15*^*+/+*^ (*n* = 20) and *Alox15*^*−/−*^ (*n* = 17) K18-hACE2 mice infected with SARS-CoV-2. Data are presented as the mean ± s.e.m. and are from one experiment (**k**,**q**), pooled from two (**a**,**c**,**e**,**f**,**m**,**p**,**s**), three (**g**,**h**,**j**,**l**,**o**) or four (**b**) independent experiments, or representative of two (n) independent experiments or six biological replicates (**d**). Data were analysed using two-tailed unpaired *t*-test (**k**), two-tailed Mann–Whitney test (**h**), one-way ANOVA followed by Tukey’s multiple comparisons test (**a**,**p**,**q**), two-way ANOVA followed by Sidak’s multiple comparisons test (**b**,**c**,**e**,**f**,**j**,**l**–**n**) or log-rank test (**o**,**s**). The models in **a**, **I**, **p** and **r** were created using BioRender (https://biorender.com). Source data

**Extended Data Fig. 6:**
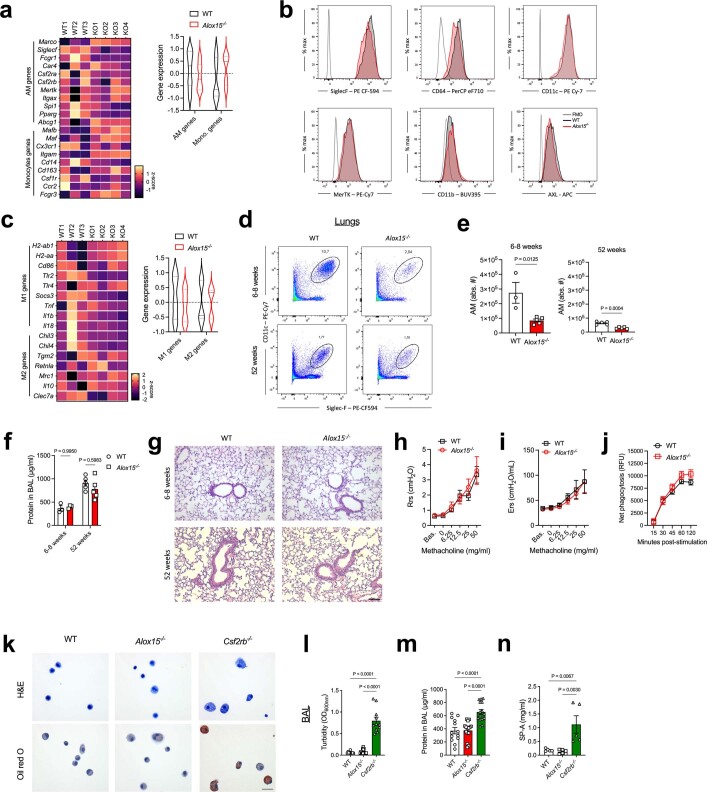
Homeostatic *Alox15*^*−/−*^ AM are similar to WT AM. (a) Heatmap and quantification of genes previously associated with alveolar macrophages or monocytes lineages (WT: n = 3, *Alox15*^*−/−*^: n = 4). (b) Representative FACS plots and quantification of surface markers expression determined by flow cytometry. (c) Heatmap and quantification of genes previously associated with alveolar macrophages phenotype (WT: n = 3, *Alox15*^*−/−*^: n = 4). (d) Representative FACS plots of AM population in 6–8 weeks and 52 weeks old WT and *Alox15*^*−/−*^ mice. (e) AM numbers in the lungs of 6–8 (WT: n = 3, *Alox15*^*−/−*^: n = 5) or 52-weeks old (n = 5/group) WT and *Alox15*^*−/−*^ mice. (f) BAL total protein levels in 6–8 (n = 3/group) or 52-weeks old (n = 5/group) WT and *Alox15*^*−/−*^ mice. (g) Representative micrographs of lung sections from naïve 6–8 weeks and 52 weeks old WT and *Alox15*^*−/−*^ mice. Bar = 100 µm. (h-i) Adult WT (n = 5) and *Alox15*^*−/−*^ (n = 6) mice were used to measure resistance (h) and elastance (i). (j) Phagocytic capacity of naïve BAL AM from adult WT and *Alox15*^*−/−*^ mice (WT: n = 7, *Alox15*^*−/−*^: n = 6 biological replicates). (k) Representative micrographs of BAL AM, cytospun, and stained with H&E (top) or Oil red O (bottom). Bar = 20 µm. (l-n) BAL was collected from adult WT, *Alox15*^*−/−*^ and *Csf2rb*^*−/−*^ mice, and BAL turbidity (l) (n = 10/14/11/group), total protein content (p) (n = 13/15/15/group) and SP-A levels (q) (n = 5/6/5/group) were measured. Data are presented as mean ± s.e.m and are from one (a, c, d-f) or pooled from two (h-j) or three (l-m) independent experiments or representative of two (b, k) independent experiments or 5 biological replicates (g). Data were analyzed using two-way ANOVA followed by Tukey’s multiple comparison test (f), two-tailed unpaired t-test (e), or one-way ANOVA followed by Tukey’s multiple comparisons test (l-n). Source data

At steady state, the transcriptomics profile and flow cytometry data showed minimum differences in genes associated with macrophage activation or polarization (Extended Data Fig. 6a–c). Despite a 50% reduction in AMs in adult (6–8 weeks) or elderly (52 weeks) *Alox15*^*−/−*^ mice (Extended Data Fig. 6d,e), total protein levels in the airways (Extended Data Fig. 6f), lung structure (Extended Data Fig. 6g) and airway mechanics and responsiveness (Extended Data Fig. 6h–i) were normal in these mice. The phagocytic capacity of *Alox15*^*−/−*^ AMs (Extended Data Fig. 6j) and surfactant catabolism were also intact (Extended Data Fig. 6k). Consistently, the turbidity of bronchoalveolar lavage (BAL) (Extended Data Fig. 6l), total BAL protein (Extended Data Fig. 6m) and surfactant protein A (SP-A) levels (Extended Data Fig. 6n) in *Alox15*^*−/−*^ mice were similar to WT mice. The levels of these parameters were also significantly less than in *Csf2rb*^*−/−*^ mice, which develop pulmonary alveolar proteinosis^11^. Furthermore, adoptive transfer of *Alox15*^*−/−*^ AMs into the lungs of *Csf2rb*^*−/−*^ recipient mice restored protein homeostasis in the alveolar space of *Csf2rb*^*−/−*^ mice (Fig. 4a). Thus, during steady state, *Alox15*^*−/−*^ AMs are able to maintain alveoli homeostasis.

**Extended Data Fig. 7:**
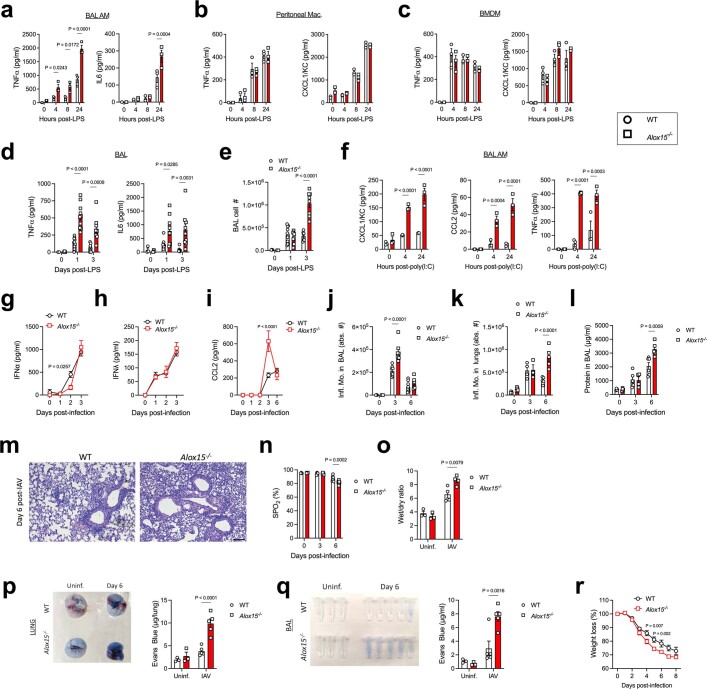
*Alox15*^*−/−*^ mice are more susceptible to acute viral infections. (a) BAL AM from adult WT and *Alox15*^*−/−*^ mice were stimulated *in vitro* using LPS (100 ng/ml) and TNFa (left) or IL6 (right) production was assessed by ELISA (n = 3 biological replicates/group). (b-c) Peritoneal macrophages (b) or BMDM (c) from adult WT and *Alox15*^*−/−*^ were stimulated with LPS (100 ng/ml) and production of TNFα and CXCL1/KC was evaluated by ELISA (n = 3 biological replicates/group). (d) TNFα and IL6 levels in BAL from adult WT and *Alox15*^*−/−*^ mice treated or not with 20 µg LPS intranasally (WT: n = 3/11/7/timepoint, *Alox15*^*−/−*^: TNFα n = 3/10/8 and IL6 3/10/9/timepoint). (e) BAL cellularity in LPS-treated (20 µg/mouse) WT and *Alox15*^*−/−*^ mice at various days post-delivery (WT: n = 3/11/7/timepoint, *Alox15*^*−/−*^: n = 3/10/8/timepoint). (f) BAL AM from adult WT and *Alox15*^*−/−*^ mice were stimulated *in vitro* using poly(I:C) (50 µg/ml) and CXCL1/KC, CCL2 and TNFα production were assessed by ELISA (n = 3 biological replicates/group). (g-o) Adult WT and *Alox15*^*−/−*^ mice were infected with IAV (50 pfu). (g-i) BAL levels of IFNα (g) (WT: n = 3/5/8/7/timepoint, *Alox15*^*−/−*^: n = 3/5/9/7/timepoint), IFNλ (h) (WT: n = 3/5/7/8 /timepoint, *Alox15*^*−/−*^: n = 3/4/7/7/timepoint) and CCL2 (i) (WT: n = 3/6/6/11/7/timepoint, *Alox15*^*−/−*^: n = 3/6/6/10/5/timepoint) at various days post-infection. (j-k) Numbers of monocytes in the BAL (j) (WT: n = 3/8/9/timepoint, *Alox15*^*−/−*^: n = 3/7/8/timepoint) and lungs (k) (WT: n = 5/4/5/timepoint, *Alox15*^*−/−*^: n = 5/3/5/timepoint) at various days post-infection. (l) Protein levels in BAL (WT: n = 4/5/6/timepoint, *Alox15*^*−/−*^: n = 4/4/5/timepoint). (m) Representative micrographs of lung sections at day 6 post-IAV infection. Bar = 100 µm. (n) Oxygen saturation at various days post-infection (WT: n = 3/5/9/timepoint, *Alox15*^*−/−*^: n = 3/5/8/timepoint). (o) Wet/dry ratio (Uninf.: n = 3/group, IAV: n = 4/group). (p-q) Representative micrographs and quantification of Evans blue extravasation in the lungs (p) and BAL (q) of IAV-infected mice at day 6 post-infection (Uninf.: n = 3/group, IAV: 5/group). (r) Representatives weight loss curves after infection with LD50 dose (90 pfu) of IAV (n = 11/group). Data are presented as mean ± s.e.m and are pooled from two (d-e, g-j, n, r) or three (a-c, f) or representative of two (k, o-q) independent experiments or 6 biological replicates (m). Data were analyzed using two-way ANOVA followed by Sidak’s multiple comparisons test (a, d-g, i-l, n-r). Source data

By contrast, *Alox15*^*−/−*^ AMs, but not BMDMs or PMs, produced significantly more tumour necrosis factor (TNF)-α, C-X-C motif chemokine 1 (CXCL1; also known as KC) and interleukin-6 (IL-6) in response to lipopolysaccharide (LPS) in vitro (Fig. 4b and Extended Data Fig. 7a–c) and in vivo (Fig. 4c and Extended Data Fig. 7d). This increase in cytokine production was associated with increased lung inflammation (Fig. 4d), tissue damage (Fig. 4e) and a significant increase in neutrophil recruitment (Fig. 4f and Extended Data Fig. 7e). Imaging of the lung microvasculature by intravital microscopy showed that in LPS-treated *Alox15*^*−/−*^ mice, LPS induced neutrophil recruitment, with significant neutrophil–neutrophil interactions leading to the formation of large, complex cellular aggregates termed clusters. This highly activated neutrophil behaviour (Fig. 4g,h and Supplementary Video 1) contributes significantly to pulmonary tissue damage^29^. Similar to LPS, *Alox15*^*−/−*^ AMs stimulated with polyinosinic:polycytidylic acid (poly(I:C)) also showed increased levels of CXCL1, C-C chemokine 2 (CCL2) and TNF-α (Extended Data Fig. 7f).

**Extended Data Fig. 8:**
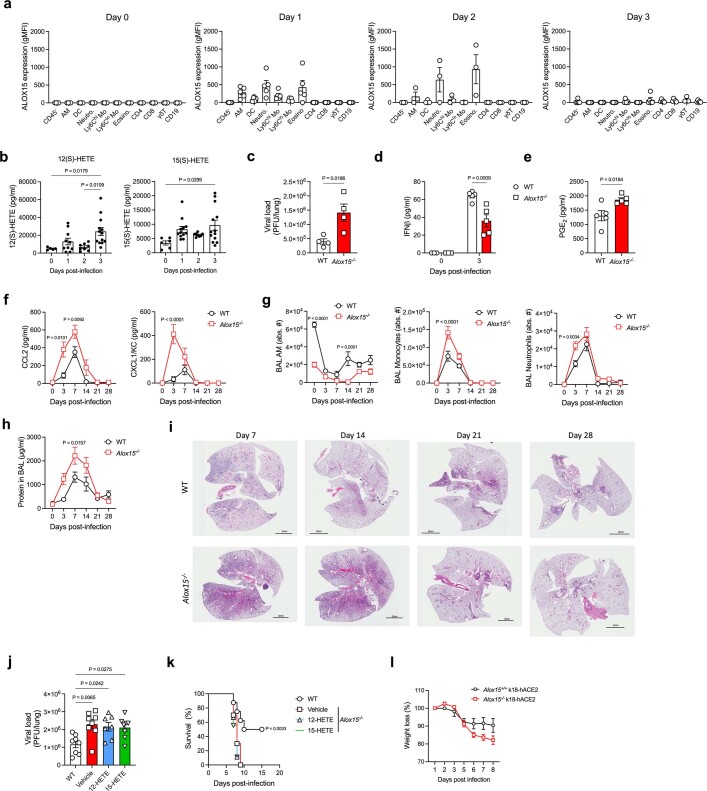
ALOX15-deficient mice are more susceptible to a low dose of IAV. (a-c) WT and *Alox15*^*−/−*^ animals were infected with IAV (50 pfu, in). (a) At various days post-infection, expression of ALOX15 was evaluated by flow cytometry (ICS) in CD45^−^ cells as well as various leukocyte populations. The expression was normalized to *Alox15*^*−/−*^ mice used as controls for gating (n = 4/5/3/5/group). (b) Levels of 12-HETE (left, n = 5/10/8/13/group) and 15-HETE (right, n = 6/12/8/13/group) in the BAL at various days post-infection. (c-i) WT and *Alox15*^*−/−*^ animals were infected with IAV (20 pfu, in). (c) Pulmonary viral loads at day 3 post-infection (n = 4/group). (d-h) BAL levels of IFNβ (d) (0: n = 3/group, 3: n = 5/group) and PGE_2_ (e) (n = 5/group) at day 3 post-infection. (f) BAL levels of CCL2 (left) and CXCL1/KC (right) at various days post-infection (WT: n = 3/5/9/5/6/3/group, *Alox15*^*−/−*^: n = 3/5/8/6/5/3 mice/group). (g) BAL numbers of AM (left), monocytes (middle) and neutrophils (right) at various days post-infection (WT: n = 3/5/5/5/6/3/timepoint, *Alox15*^*−/−*^: n = 3/5/5/6/5/3/timepoint). (h) Total BAL protein levels at various days post-infection (WT: n = 3/5/9/5/6/3/group, *Alox15*^*−/−*^: n = 3/5/7/6/5/3/group). (i) Representative micrographs of H&E lung sections at various days post-infection. (j) *Alox15*^*−/−*^ mice were treated daily with exogenous 12-HETE or 15-HETE or vehicle. Lungs were harvested at day 3 post-infection for assessment of pulmonary viral loads (n = 8/8/7/8/group). (k) *Alox15*^*−/−*^ mice were treated daily with exogenous 12-HETE or 15-HETE or vehicle starting on the day of infection (IAV, 90 pfu, in) for 3 days. The survival was monitored overtime (n = 8/10/9/9/group). (l) Weight loss curves after SARS-CoV-2 infection (k18-hACE2 *Alox15*^*+/+*^: n = 19, k18-hACE2 *Alox15*^*−/−*^: n = 17). Data are presented as mean ± s.e.m and are from one (a, c-e, k) or pooled from two (b, f-h, j, l) independent experiments or representative of 5 (i) biological replicates. Data were analyzed using two-tailed unpaired t-test (c, e), one-way ANOVA followed by Tukey’s multiple comparisons test (b, j), two-way ANOVA followed by Sidak’s multiple comparisons test (d, f-h) or log-rank test (k). Source data

Considering that senescent *Alox15*^*−/−*^ AMs produced more PGE_2_, and we have previously shown that PGE_2_ is detrimental during influenza A virus (IAV) infection^14^, we infected *Alox15*^*−/−*^ mice with IAV (Fig. 4i). The production of PGE_2_ in *Alox15*^*−/−*^ AMs infected with IAV in vitro, ex vivo and in vivo was significantly increased (Fig. 4j–l). In line with the PGE_2_-mediated suppression of early IFN-I production during IAV infection^14^, the production of IFN-I, IFNβ (Fig. 4m) and IFNα (Extended Data Fig. 7g), but not IFN-III or IFNλ (Extended Data Fig. 7h), was significantly reduced in the BAL at day 2 after infection. A delay in the early IFN-I response resulted in increased pulmonary viral loads at day 3 after infection (Fig. 4n). This was associated with a significant increase in the production of CCL2 (Extended Data Fig. 7i), an accumulation of pulmonary inflammatory monocytes (Extended Data Fig. 7j,k), lung damage (Extended Data Fig. 7l–q) and an increase in mortality (Fig. 4o and Extended Data Fig. 7r). The expression of ALOX15 was not detectable in the lung at steady state (Extended Data Fig. 8a). However, following IAV infection, different cell types showed detectable levels of ALOX15 expression at day 1 and day 2, including AMs, neutrophils and eosinophils. Levels were undetectable by day 3 after IAV infection (Extended Data Fig. 8a). Concomitantly, the production of 12-HETE and 15-HETE in the BAL of IAV-infected mice was significantly increased at day 3 after infection (Extended Data Fig. 8b). Similar to the results obtained with 50 plaque-forming units (p.f.u.) of IAV, *Alox15*^*−/−*^ animals infected with a low dose of IAV (20 p.f.u.), which causes no mortality, displayed an increase in pulmonary viral load, PGE_2_, CXCL1, CCL2, neutrophils and inflammatory monocytes and a decrease in IFNβ (Extended Data Fig. 8c–g). This led to increased alveolar damage (Extended Data Fig. 8h) and pulmonary inflammation (Extended Data Fig. 8i). Thus, the lack of ALOX15 pathway has a significant impact on the initial antiviral response, which in turn dictates the magnitude of the inflammatory responses.

Notably, treatment of *Alox15*^*−/−*^ with 12-HETE or 15-HETE had no effect on the pulmonary viral load or the outcome of IAV infection (Extended Data Fig. 8j,k). However, adoptive transfer of WT AMs into the airways of *Alox15*^*−/−*^ mice (Fig. 4p) or inhibition of PGE_2_ production in *Alox15*^*−/−*^ mice (Fig. 4q) significantly reduced the pulmonary viral load. These data suggest that impaired function of *Alox15*^*−/−*^ AMs is responsible for the susceptibility of the host to IAV infection, which cannot be reversed by exogenous 12-HETE or 15-HETE. Finally, we generated *Alox15*^*−/−*^ K18-hACE2 mice and infected them with a sublethal dose of SARS-CoV-2 (Fig. 4r). *Alox15*^*−/−*^ K18-hACE2 mice were highly susceptible to infection, with almost 100% mortality (Fig. 4r,s and Extended Data Fig. 8l). Thus, impaired proliferation and increased senescence in *Alox15*^*−/−*^ AMs leads to increased susceptibility to acute pulmonary viral infections.

## AM programming by neutrophils

**Fig. 5:**
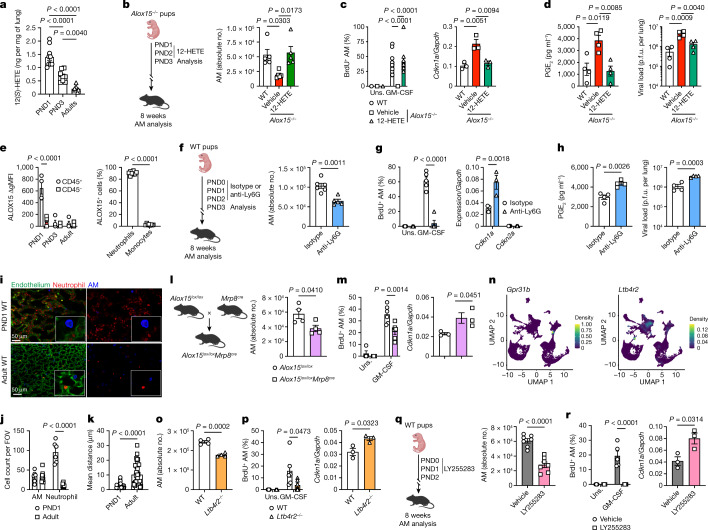
Neonatal neutrophil-derived 12-HETE programmes AM proliferation. **a**, Pulmonary levels of 12(*S*)-HETE in PND1, PND3 and adult WT mice (left to right, *n* = 10, 8 or 8 per group). **b**, Scheme (left) and BAL AM numbers (right) in 12-HETE-treated *Alox15*^*−/−*^ pups that were left to age until adulthood (*n* = 5 per group). **c**, Left, BrdU^+^ AMs after GM-CSF culture (*n* = 5 (Uns.) or 11, 10 or 12 (GM-CSF) fields of view (FOVs) per group). Right, basal *Cdkn1a* expression in AMs (*n* = 3 biological replicates per group). **d**, BAL PGE_2_ levels (left) and pulmonary viral loads (right) at day 3 after IAV infection (50 p.f.u.) (*n* = 4 per group). **e**, Left, quantification of ALOX15 expression in CD45^+^ or CD45^−^ lung cells at various ages (*n* = 4 mice per group). MFI, mean fluorescence intensity. Right, ALOX15^+^ CD45^+^ cells were further gated using CD11b, Ly6C and Ly6G in PND1 lungs (*n* = 8 mice per group). **f**, Postnatal neutrophil depletion in WT mice using anti-Ly6G (left) and quantification (right) of BAL AMs in animals that were left to age until adulthood (*n* = 6 (isotype) or 5 (anti-Ly6G) mice per group). **g**, Left, BrdU^+^ AMs after GM-CSF culture (*n* = 3 (Uns.) or 5 (GM-CSF) fields of view per group). Right, basal *Cdkn1a* and *Cdkn2a* expression in AMs (*n* = 3 biological replicates per group). **h**, BAL PGE_2_ levels (left) and pulmonary viral loads (right) at day 3 after IAV infection (50 p.f.u.) (*n* = 4 mice per group). **i**, Lungs were isolated from PND1 or adult (6–8 weeks) WT mice and stained for Ly6G (neutrophils, red), CD11c (AMs, blue) and CD31 (endothelial cells, green). **j**, Quantification of macrophages and neutrophils (PND1: n = 6, Adults: n = 11). **k**, Mean distance between neutrophils and macrophages (PND1: n = 45, Adult: n = 43). **l**, Left, generation of neutrophil-specific *Alox15*^*lox/lox*^*Mrp8*^*cre*^ mouse model. Right, BAL AM numbers in adult mice (*n* = 4 per group). **m**, Left, BrdU^+^ AMs after GM-CSF culture (*n* = 4 (Uns.) or 6 (GM-CSF) fields of view). Right, basal *Cdkn1a* expression (*n* = 3 biological replicates per group). **n**, Representative uniform manifold approximation and projection (UMAP) plots of *Gpr31b* and *Ltb4r2* expression by single-cell RNA-seq in PND1 lungs. **o**, BAL AM numbers in adult WT and *Ltb4r2*^*−/−*^ mice (*n* = 4 per group). **p**, BrdU^+^ AMs after GM-CSF culture (left; *n* = 3 (Uns.) or 6 (GM-CSF) fields of view per group) and basal *Cdkn1a* expression (right; *n* = 3 biological replicates per group) in adult WT and *Ltb4r2*^*−/−*^ AMs. **q**, Postnatal inhibition of LTB4R2 signalling using LY255283 in WT mice (left) and BAL AM numbers in adult mice (right) (*n* = 7 per group). **r**, BrdU^+^ AMs after GM-CSF culture (left; *n* = 3 (Uns.) or 5 (GM-CSF) fields of view per group) and basal expression of *Cdkn1a* (right; *n* = 3 biological replicates per group) in adult AMs. Data are presented as the mean ± s.e.m. and are pooled from one (**d**,**h**,**n**), two (**a**,**b**,**e**,**f**,**l**,**o**,**q**) or three (**c**,**g**,**i**–**k**,**m**,**p**,**r**) independent experiments or representative of two (**c**,**g**,**m**,**p**,**r** (left)) independent experiments and were analysed using two-tailed unpaired *t*-test (**e**–**h**,**k**–**m**,**o**–**r**), one-way ANOVA followed by Tukey’s multiple comparisons test (**a**–**d**) or two-way ANOVA followed by Tukey’s (**c**) or Sidak’s (**e**,**g**,**j**,**m**,**p**,**r**) multiple comparisons test. The models in **b**, **f**, **l** and **q** were created using BioRender (https://biorender.com). Source data

**Extended Data Fig. 9:**
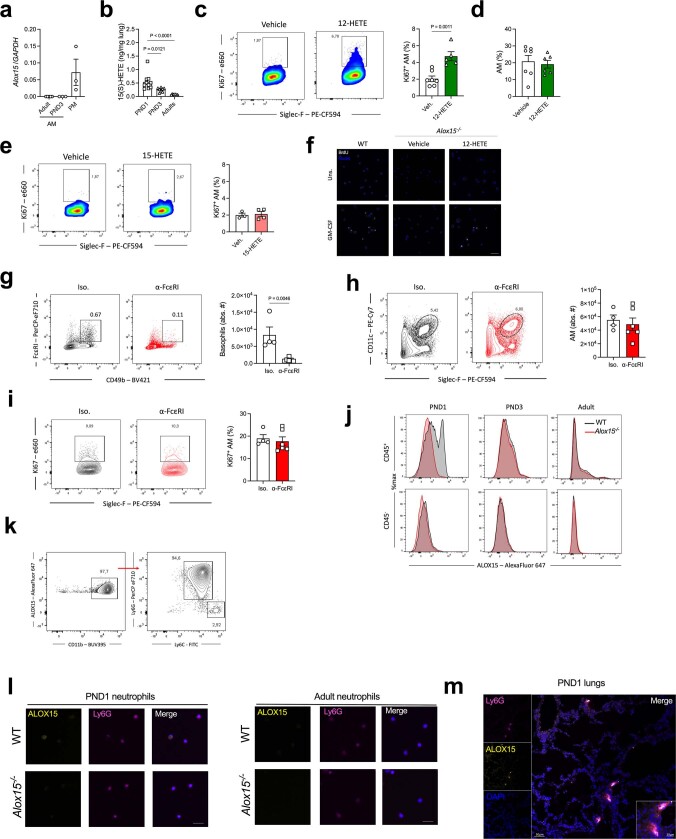
12-HETE-treated pups have restored proliferation. (a) *Alox15* expression was measured in BAL AM (PND3 and adult mice) or peritoneal macrophages using qPCR (n = 5/3/3 biological replicates/group). (b) Pulmonary levels of 15(S)-HETE in PND1, PND3 and adult WT mice (n = 10/8/8/group). (c) Representative FACS plots and quantification of Ki67^+^ AM after 12-HETE treatment (Veh.: n = 7, 12-HETE: n = 5). (d) AM populations after 12-HETE treatment (Veh.: n = 7, 12-HETE: n = 6). (e) Representative FACS plots and quantification of Ki67^+^ AM after 15-HETE treatment (Veh.: n = 3, 15-HETE: n = 4). (f) Representative micrographs of BrdU staining *in vitro* after AM culture with GM-CSF. Bar=50 µm. (g-i) PND1 WT pups were delivered intranasally anti-FcεR1 antibodies or isotype controls for two consecutive days. (g) Basophil depletion, (h) AM populations and (i) Ki67^+^ AM were determined at PND3 (Iso.: n = 4, α-FcεRI: n = 6). (j) Representative histograms of ALOX15 expression in CD45^+^ or CD45^−^ lung cells at various ages of WT and *Alox15*^*−/−*^ mice. (k) PND1 ALOX15^+^ CD45^+^ cells were further gated using CD11b, Ly6C and Ly6G. (l) Lung and blood neutrophils were isolated from PND1 or adults (6–8 weeks) WT or *Alox15*^*−/−*^ mice. Expression of ALOX15 and Ly6G were assessed by immunofluorescence. Blue: DAPI. Bar = 20 µm. (m) Representative micrographs of ALOX15 and Ly6G expression in frozen lung sections of PND1 WT pups. Data are presented as mean ± s.e.m and are from one (g-i) or pooled from two (c-e) or three (a-b) independent experiments or representative from two (f, j, k, l, m) independent experiments. Data were analyzed using two-tailed unpaired t-test (c, g) or one-way ANOVA followed by Tukey’s multiple comparisons test (b). Source data

ALOX15-deficient AMs exhibit impaired proliferation, even though *Alox15* is not expressed in mouse adult lungs^30^ or by AMs themselves at steady state (Extended Data Fig. 9a). Therefore we asked whether a burst of ALOX15 occurs during postnatal lung development. ALOX15-derived 12(*S*)-HETE and 15(*S*)-HETE were significantly increased in the lungs of WT mice at PND1 compared with PND3 and gradually decreased with age (Fig. 5a and Extended Data Fig. 9b). Moreover, delivery of exogenous 12-HETE, but not 15-HETE, at PND1 and PND2 significantly increased the proliferation of AMs, but had no effect on their differentiation (Extended Data Fig. 9c–e). The effects of perinatal 12-HETE treatment in *Alox15*^*−/−*^ mice was long term, as the number of AMs in the BAL was maintained and comparable to WT in adult mice (Fig. 5b). Notably, AMs from prenatal 12-HETE-treated *Alox15*^*−/−*^ mice restored their proliferative capacity with decreased expression of the senescence marker *Cdkn1a* (Fig. 5c and Extended Data Fig. 9f). Functionally, IAV-infected 12-HETE-treated *Alox15*^*−/−*^ mice showed significantly lower levels of PGE_2_ in the BAL, which was associated with a reduced viral load (Fig. 5d). Thus, 12-HETE is required for imprinting the self-renewal capacity of AMs during the differentiation of fetal liver monocytes into AMs to prevent senescence and restore the antimicrobial capacity of AMs.

**Extended Data Fig. 10:**
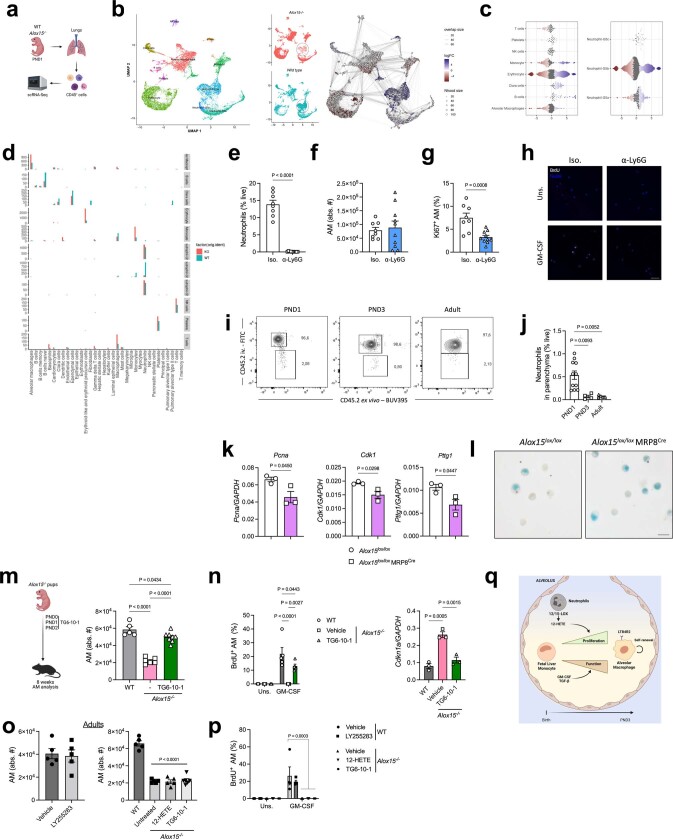
Neonatal neutrophils program AM proliferative capacity. (a) CD45^+^ cells were isolated from the lungs of PND1 WT and *Alox15*^*−/−*^ pups and subjected to scRNA-Sequencing. (b) Uniform Manifold Approximation and Projection (UMAP) embedding of integrated CD45^+^ cells from the lungs of WT and *Alox15*^*−/−*^ mice. Unsupervised clusters were labelled using CelliD^50^ and the expression levels of canonical cell type markers. Neutrophils were further partitioned through label transfer from adult mouse neutrophil cell atlas to create a final assignment (left). A graph representation of the results from Milo differential abundance testing (right). Nodes are neighbourhoods, coloured by their log fold change between WT and *Alox15*^*−/−*^ cells. Non-DA neighbourhoods (FDR > 10%) are coloured white, and sizes correspond to the number of cells in a neighbourhood. There is a marked increase in the relative abundance of WT cells in the “Neutrophil-G5b” cluster compared to *Alox15*^*−/−*^ cells. (c) Beeswarm plot showing the distribution of log-fold change between WT and *Alox15*^*−/−*^ cells in neighborhoods containing cells from different cell type clusters. Blue refers to neighborhoods within particular cell types that are significantly enriched (FDR<5%) in WT compared to *Alox15*^*−/−*^ animals and brown neighborhoods enriched in *Alox15*^*−/−*^ animals relative to WT. (d) Cell type label frequencies from PanglaoDB predictions within finalized manual labeling clusters (vertical facets) and split by condition. Confirms finalized labels as consistent with automated predictions. (e-g) quantification of neutrophils (e), total (f) and Ki67^+^ (g) alveolar macrophages in the lungs of PND3 WT mice following isotype or anti-Ly6G treatment (Iso.: n = 8, α-Ly6G: n = 10). (h) Representative micrographs of BrdU staining *in vitro* after AM culture with GM-CSF. Bar = 50 µm. (i-j) Parenchymal neutrophils were determined in WT mice at various ages by intravascular and *ex vivo* CD45.2 staining. Representative FACS plots (i) and quantification (j) (n = 11/4/5/group). (k) Expression of various genes involved in proliferation in BAL AM from adult *Alox15*^*lox/lox*^ and *Alox15*^*lox/lox*^ MRP8^Cre^ (n = 3 biological replicates/group). The genes were previously identified in our bulk RNA-Seq dataset on whole body *Alox15*^*−/−*^ mice. (l) Representative micrographs of SA-β-galactosidase in BAL AM from *Alox15*^*lox/lox*^ and *Alox15*^*lox/lox*^ MRP8^Cre^ mice. Bar=10 µm. (m) Model of EP2 signaling inhibition with TG6-10-1 in pups (left) and BAL AM numbers in adult mice (right) (n = 5/6/8/group). (n) BrdU^+^ AM following GM-CSF culture *in vitro* (left) (Uns.: n = 3, GM-CSF: n = 5 fields of view/group) and basal *Cdkn1a* expression (right) (n = 3 biological replicates/group) in BAL AM from adult mice. (o-p) Adult WT mice were treated with LY255283 and adult *Alox15*^*−/−*^ mice were treated with TG6-10-1 or 12-HETE for three consecutive days. AM populations in the BAL were evaluated after 7 days resting period (o) (left, n = 5/group; right, WT: n = 5, Untreated: n = 5, 12-HETE: n = 5, TG6-10-1: n = 6) and BrdU^+^ AM after 3 days of culture with GM-CSF *in vitro* (p) (Uns.: n = 3, GM-CSF: n = 4 fields of view/group). Data are presented as mean ± s.e.m and are from one (a-d, o-p) or pooled from two (e-g, i-j, m) or three (k, n) independent experiments or representative of two (h, l, n, p) independent experiments. Data were analyzed using two-tailed unpaired t-test (e, g, k), one-way ANOVA followed by Tukey’s multiple comparison test (j, m-o) or two-way ANOVA followed by Sidak’s multiple comparisons test (n, p). The models in (a, m, q) were created using BioRender (https://biorender.com). Source data

To identify the source of 12-HETE in the neonatal lung, we first depleted basophils, which have been shown to express ALOX15 in the neonatal lung and play an important part in the differentiation of AMs^31^. However, basophil depletion in WT mice affected neither the seeding of AMs nor their proliferation (Extended Data Fig. 9g–i). We next analysed the expression of ALOX15 in leukocytes (CD45^+^) or structural cells (CD45^−^) in the lungs of PND1, PND3 and adult mice by flow cytometry (Fig. 5e and Extended Data Fig. 9j,k). The number of ALOX15-expressing leukocytes, in particular neutrophils (CD11b^+^Ly6G^+^), was significantly increased in the lungs of PND1 mice, but not adult lungs (Fig. 5e and Extended Data Fig. 9l,m). We next performed single-cell RNA-seq on isolated CD45^+^ cells from the lungs of WT and *Alox15*^*−/−*^ mice at PND1. This analysis revealed marked compositional differences in neutrophil populations between *Alox15*^*−/−*^ mice and WT mice (Extended Data Fig. 10a–d). *Alox15*^*−/−*^ mice showed an almost complete absence of an entire subpopulation of neutrophils that represented 63% of all mature neutrophils found in WT mice (Extended Data Fig. 10b,c). By comparison, the compositional differences detected among other cell types were negligible, which further implicates neutrophils in the programming of AMs. Indeed, neutrophil depletion from PND0 to PND2 significantly reduced the proliferative capacity of AMs, but not seeding, at PND3 (Extended Data Fig. 10e–g). This in turn led to reduced numbers of AMs in adult mice (Fig. 5f), which was due to decreased proliferation of AMs and increased expression of the senescence marker *Cdkn1a* (Fig. 5g and Extended Data Fig. 10h). Of note, this was associated with increased PGE_2_ production and pulmonary viral load following IAV infection (Fig. 5h). Whole-lung imaging and intravascular staining showed that neutrophils specifically accumulated in the lung parenchyma of PND1 mice (Fig. 5i,j and Extended Data Fig. 10i,j). Moreover, the distance between AMs and neutrophils in the neonatal lungs was significantly reduced compared with adult lungs of WT mice (Fig. 5k). This result suggests a potential interaction between neutrophils and AMs in the early days of life. Finally, we generated a conditional knockout strain in which *Alox15* is selectively deleted in neutrophils by crossing *Alox15*^*lox/lox*^ mice with *Mrp8*^*cre*^ mice (Fig. 5l). Similar to *Alox15*^*−/−*^ mice, *Alox15*^*lox/lox*^*Mrp8*^*cre*^ mice had significantly fewer numbers of AMs in the BAL, which was associated with impaired proliferation, increased expression of *Cdkn1a* and β-galactosidase (Fig. 5l,m and Extended Data Fig. 10k,l).

Two receptors have been identified for 12-HETE: GPR31 (encoded by *Gpr31b*)^32^ and LTB4R2 (encoded by *Ltb4r2*)^33^. Our single-cell RNA-seq datasets showed no detectable *Gpr31b* expression, whereas there was a strong expression of *Ltb4r2* in PND1 macrophages (Fig. 5n). Similar to *Alox15*^*−/−*^ mice, *Ltb4r2*^*−/−*^ mice had significantly fewer numbers of AMs in the BAL at steady state (Fig. 5o). The reduced numbers of AMs were associated with a decrease in their proliferation and an increase in the expression of *Cdkn1a* in *Ltb4r2*^*−/−*^ mice (Fig. 5p). We next treated WT mice at PND0, PND1 and PND2 with a pharmacological antagonist of LTB4R2 (LY255283) and mice were left to age until adulthood (Fig. 5q). AMs from mice that were treated with LY255283 perinatally had significantly fewer AMs in the BAL (Fig. 5q). This was associated with a significant decrease in proliferation of AMs and an increase in the expression of the cell cycle inhibitor *Cdkn1a* (Fig. 5r). An analysis of the AM populations and proliferation after blocking PGE_2_ signalling with an EP_2_ antagonist (TG6-10-1) in *Alox15*^*−/−*^ mice showed that the number of AMs in the BAL was significantly increased, reaching a level similar to that of WT mice (Extended Data Fig. 10m). This was associated with an increase in proliferation and a decrease in *Cdkn1a* expression in AMs (Extended Data Fig. 10n). These processes were specific to the perinatal window, as no effect was observed in adult mice (Extended Data Fig. 10o,p). Collectively, these data indicate that perinatal ALOX15-derived 12-HETE from neutrophils is required to imprint the long-term self-renewing programme of AMs through the LTB4R2 receptor.

Since the advent of fate-mapping approaches demonstrating the prenatal origins of some RTMs, the cellular and molecular mechanisms of self-renewing, embryo-derived macrophages have remained elusive. Although GM-CSF and TGFβ are required for the differentiation of fetal liver monocytes to mature AMs during the first days of lung development^2,9^, our results demonstrated that these cytokines are not sufficient for RTM self-renewal and maintenance. Specifically, we showed that neutrophil-derived 12-HETE during early life is essential for the programming of AM self-proliferation, revealing a new role for innate immunity and eicosanoids during the perinatal period (Extended Data Fig. 10q). It is unclear whether similar mechanisms of reprogramming are engaged to replace AMs by BMDMs. Furthermore, the location of this imprinting (for example, BM or lung) remains unknown. Although ageing is associated with alterations in haematopoiesis and  increased numbers of circulating monocytes and neutrophils, the number of AMs declines with age^34,35^. The AM reduction in ageing individuals and their increased susceptibility to pulmonary infection or inflammation may be related to the attenuation of neutrophil function with age^36^, including their reduced capacity to produce 12-HETE. Although we did not investigate the origin of neutrophils (BM compared to fetal liver haemopoietic stem cells) during prenatal lung development, the increased number of neutrophils in the lung parenchymal tissue raises questions on the specific age-dependent origin of neutrophils that might still derive from fetal haematopoiesis. Further studies are required to examine the ontogeny and functions of neutrophils with age and the therapeutic potential of eicosanoid pathways in modulating the quantity or quality of AMs during pulmonary infection, resolution of inflammation and mucosal healing.

## Methods

### Mice

C57BL/6 mice, *Alox15*^*−/−*^ mice (B6.129S2-*Alox15*^*tm1Fun*^/J), *Alox5*^*−/−*^ mice (B6.129S2-*Alox5*^*tm1Fun*^/J), CD45.1 mice (B6.SJL-*Ptprc*^*a*^ *Pepc*^*b*^/BoyJ), *Mrp8*^*cre*^ mice (B6.Cg-Tg(S100A8-cre,-EGFP)1Ilw/J), *Csf2rb*^*−/−*^ mice (B6.129S1-*Csf2rb*^*tm1Cgb*^/J) and K18-hACE2 (B6.Cg-Tg(K18-ACE2)2Prlmn/J) mice were purchased from Jackson Laboratories. *Alox15*^*−/−*^ K18-hACE2 mice were generated by crossing K18-hACE2 mice with *Alox15*^*−/−*^ animals. *Alox15*^*lox/lox*^ mice on a C57BL/6 background were a gift from S. Tersey (University of Chicago, USA) and were crossed with *Mrp8*^*cre*^ mice to generate specific deletion of the ALOX15 pathway in neutrophils. *Ltb4r2*^*−/−*^ mice on a C3HeJ background were a gift from C. Brown (University of Missouri, USA). All animals were housed and inbred at the animal facility of the Research Institute of McGill University under specific pathogen-free conditions with ad libitum access to food and water, a temperature of 21 °C (±1 °C), relative humidity of 40–60% (±5%) and light cycle of 12 h on, 12 h off (daily cycle). Mice were randomly allocated to experimental groups, and experiments were performed using both female and male age- and sex-matched mice.

### Isolation and culture of primary macrophages and cell lines

AMs were collected by BAL of naive mice using cold, sterile PBS (5 × 1 ml for adult mice; 5 × 50–100 µl for PND3). AMs were cultured in RPMI-1640 medium supplemented with 10% (v/v) FBS, 2 mM l-glutamine, 10 mM HEPES and 100 U ml^–1^ penicillin–streptomycin. After 1 h of adhesion, AMs were washed with PBS and placed in fresh medium. Mouse BMDMs were isolated following aseptic flushing of tibiae and femurs of 6–8-week-old mice. Macrophages were differentiated from BM precursors for 6 days in RPMI-1640 supplemented with 30% (v/v) L929 cell-conditioned (American Type Culture Collection (ATCC)) medium, 10% (v/v) FBS, 2 mM l-glutamine, 1 mM sodium pyruvate, 1% essential and nonessential amino acids, 10 mM HEPES and 100 U ml penicillin–streptomycin. PMs were collected by peritoneal lavage of naive mice using cold, sterile PBS. PMs were cultured in RPMI-1640 supplemented with 10% (v/v) FBS, 2 mM l-glutamine, 10 mM HEPES and 100 U ml^–1^ penicillin–streptomycin. After 1 h of adhesion, PMs were washed with PBS and placed in fresh medium. All reagents and supplements pertaining to cell culture were purchased from Gibco.

### Viruses, infections and stimulation

All influenza in vitro and in vivo infections were performed using influenza A/Puerto Rico/8/34 (H1N1) virus, which was provided by J. A. McCullers (St Jude Children Research Hospital). Virus was propagated and titrated in MDCK (ATCC) cells using standard plaque assay^37^. Mice were intranasally challenged (in 25 μl PBS) with IAV at a sublethal dose of 20 or 50 p.f.u. (immunophenotyping, ELISA) or a median lethal dose (LD_50_) of 90 p.f.u. In some experiments, *Alox15*^*−/−*^ mice were treated intraperitoneally with a chemical inhibitor of mPGES1 (CAY10526, 5 mg kg^–1^, in 100 µl of PBS, daily) or exogenous 12-HETE or 15-HETE (1 µg per mouse in 100 µl of PBS) starting from the day of infection. Virus titres were determined in lung homogenates homogenized in PBS using standard MDCK plaque assay.

For SARS-CoV-2 infection in vivo, SARS-CoV-2/RIM-1 was isolated from a patient at The McGill University Health Center, Montreal, Quebec. SARS-CoV-2 was propagated in VeroE6 cells (ATCC). Mice were intratracheally infected as previously described^38^ with 4,000 median tissue culture infectious dose (TCID_50_) of SARS-CoV-2/SB2.

In specific experiments, AMs, PMs or BMDMs were stimulated with 100 ng ml^–1^ of LPS (Sigma) or AMs were stimulated with 50 µg ml^–1^ of poly(I:C) (Invivogen).

### Protein in BAL

BAL samples collected by cannulating the trachea with a 22-gauge cannula, then washing the lungs with 3× 1 ml of cold, sterile PBS. The total volume of the recovered fluid after lavage was around 0.7 ml. Samples were centrifuged (1,500 r.p.m., 10 min), and total protein content was assessed using a Pierce BCA Protein assay (ThermoFisher).

### Wet-to-dry ratio

Lungs were collected from naive or IAV-infected mice (50 p.f.u., day 6 after infection) and blood clots were carefully removed. Then the lungs were weighed (wet weight), dried in an oven (56 °C, 2 days) and the dry weight was measured. Data are presented as the ratio wet/dry (w/w).

### Flow cytometry

Lung tissues were perfused with 10 ml of PBS, collected and minced before collagenase IV digestion (150 U ml^–1^, Sigma) for 1 h at 37 °C. Lungs were filtered through a 70 µm nylon mesh, and red blood cells were lysed. Peritoneal cells were obtained following lavage with 5 ml of cold PBS intraperitoneally injected. Cells were then spun down, and red blood cells were lysed. Spleen cells were obtained by crushing the spleen through a 70 µm nylon mesh followed by red blood cells lysis. Liver cells (median lobe) were obtained after mincing and digestion with collagenase VIII (1 mg ml^–1^, Sigma) for 30 min at 37 °C. The cells were passed sequentially through 100 and 70 µm cell strainers before red blood cell lysis. Brain cells were obtained after passing through 100 and 70 µm cell strainers followed by Percoll gradient (30% and 70% solutions). Total lung, peritoneal, liver, brain and spleen cell counts were determined with a haemocytometer, and 1–2 million cells were used for staining.

Cells were initially stained with viability dye e450 or e506 (Invitrogen, 20 min, 4 °C) and surface stained with anti-CD16/32 (BD Bioscience) in 0.5% BSA/PBS solution to block nonspecific AB interaction with Fc receptors (10 min, 4 °C). Cells were then surface-stained with different combinations of PE-Cy7-conjugated anti-CD11c, PE-CF594-conjugated anti-Siglec-F, PE-Cy7-conjugated or BUV395-conjugated anti-CD11b, PerCP-eFluor710-conjugated anti-Ly6G, FITC-conjugated or APC-conjugated anti-Ly6C, APC-eFluor780-conjugated anti-F4/80, PE-conjugated anti-CD103, PerCP-conjugated anti-CD64, BUV395-conjugated anti-CD45.2 and APC-conjugated anti-CD45.1 (all from BD Biosciences). For Ki-67, p53, pSTAT5, pSMAD2/3, p-p38, pAKT and pERK1/2, cells were fixed and permeabilized using BD CytoFix/CytoPerm (BD Bioscience) before intracellular staining with APC-conjugated or PE-conjugated anti-Ki-67, PE-conjugated anti-p53, AlexaFluor647-conjugated anti-p-p38, PE-conjugated anti-pSMAD2/3, APC-conjugated anti-pAKT1, PerCP-eFluor710-cojugated anti-pERK1/2, PE-Cy7-conjugated anti-p-γH2AX, AlexaFluor 647-conjugated anti-15-lipoxygenase and APC-conjugated anti-pSTAT5 (from BD Bioscience, Life Technologies, Bioss or Cell Signaling Technologies). In some experiments, AMs were stained with MitoSox (Invitrogen) for analysis of mitochondrial reactive oxygen species production or DAPI (1:2,000 in PBS) to evaluate the total DNA content. Flow cytometry was performed using a BD LSR Fortessa X-20 instrument (BD Biosciences) with FACSDiva software v.8.0.1 (BD Biosciences). Analysis was performed using FlowJo software v.10.7.1 (Tree Star).

### Cell death analysis

Necrosis and apoptosis levels of BAL AMs were assessed using a PE-AnnexinV and 7-amino-actinomycin D (7-AAD) Apoptosis Detection Kit I (BD Biosciences) according to the manufacturer’s instructions and analysed by flow cytometry.

### BrdU incorporation studies

For in vivo analysis, BrdU was intraperitoneally administered (1 mg per 100 µl per mouse) daily for 7 days before euthanasia. BrdU incorporation in AMs and PMs was assessed using a BrdU APC kit (BD) following the manufacturer’s instructions before analysis by flow cytometry.

For in vitro analysis, BrdU (10 µM) was added to AMs on day 2 after GM-CSF (20 ng ml^–1^) stimulation. On day 3, AMs were fixed with 4% paraformaldehyde (PFA) and then DNA was denatured using 1.5 M HCl for 30 min at room temperature. Cells were washed with PBS then stained with anti-BrdU antibody (BioLegend). BrdU (10 µM) was added to cells at day 3 and day 5 of BMDM differentiation. BrdU incorporation was assessed by flow cytometry at day 6 using a BrdU APC kit (BD) following the manufacturer’s instructions.

### Histopathological analysis

Lungs were inflated and fixed for 48 h with 10% formalin, and then embedded in paraffin. Sections (5 μm) were cut and stained with haematoxylin and eosin. Slides were scanned at a resolution of ×40 magnification, and pictures were taken using a Leica Aperio slide scanner (Leica).

For frozen sectioning and ALOX15 and Ly6G staining, lungs were inflated with 10% OCT in PBS and embedded in OCT before being frozen at −80 °C. Sections (5 µm) were dried on slides and fixed for 5 min in ice-cold acetone:methanol solution (1:3, v/v) and then washed in PBS. Lung sections were incubated for 1 h with cold block buffer (2% BSA diluted in PBS with 1:100 Fc-block). Sections were then incubated with AlexaFluor 594-conjugated anti-Ly6G (BioLegend) or a rabbit anti-ALOX15 antibody (Abcam) for 24 h in 2% BSA in PBS. Slides were then incubated with a goat anti-rabbit AlexaFluor 647-conjugated antibody (Life Technologies) for 1 h at room temperature. Slides were washed with PBS and mounted in Prolong Diamond antifade with DAPI (Invitrogen). Images were acquired using a Zeiss LSM 700 laser-scanning confocal microscope.

For whole-lung imaging of adult or PND1 mice, animals were euthanized, and 1.5% agarose was used to fill the lungs. The trachea was opened, and agarose was added to the lungs using a 25-gauge needle syringe. Lungs were removed and fixed using 4% PFA overnight and sliced into 300 µm sections using a vibratome. Lungs were stained using AlexaFluor 647-conjugated anti-mouse CD11c, AlexaFluor 594-conjugated anti-mouse Ly6G and AlexaFluor 488-conjugated anti-mouse CD31 fluorescent antibodies (all from BioLegend). Samples were mounted on slides and imaged using a Leica SP8 confocal microscope.

### Confocal microscopy

AMs were seeded in a media chamber of a glass microscopy slide (Millipore). Cells were fixed in 4% PFA for 15 min and then permeabilized by incubating with 0.1% Triton X-100 in PBS or ice-cold methanol for 15 min. Samples were blocked with 1% milk in PBS Triton X-100 0.1% for 1 h and then incubated with a specific rabbit polyclonal anti-pSTAT5, rat anti-Ki-67 or mouse anti-BrdU overnight at 4 °C (Cell Signaling Technology, Life Technologies or BioLegend). Cells were incubated for 1 h with secondary antibody Alexa Fluor 555-conjugated or 647-conjugated goat anti-rabbit or anti-mouse (1:1,000, Invitrogen) and nuclei were stained with DAPI (1:2,000, Molecular Probes). Coverslips were mounted (ProLong Diamond Anti Fade, Invitrogen) onto microscope slides. Images were acquired using a Zeiss LSM 700 laser-scanning confocal microscope and analysed using ImageJ software.

In some experiments, lung and blood neutrophils from PND1 and adult WT or *Alox15*^*−/−*^ mice were isolated using a neutrophil enrichment kit (StemCell Technologies). Pooled neutrophils were seeded onto poly-l-lysine-treated microscopy chambers and fixed with 4% PFA at room temperature for 15 min. Cells were then permeabilized using Triton X-100 0.1% and then incubated with a rabbit anti-ALOX15 antibody (Abcam) for 24 h in 2% BSA in PBS. Slides were then incubated with AlexaFluor 594-conjugated anti-Ly6G (BioLegend) and a goat anti-rabbit AlexaFluor 647-conjugated antibody (Life Technologies) for 1 h at room temperature. Slides were washed with PBS and mounted in Prolong Diamond antifade with DAPI (Invitrogen). Images were acquired using a Zeiss LSM 700 laser-scanning confocal microscope.

### ELISA and cytokine array

IFNβ and IFNα levels in BAL were measured using a Verikine Mouse IFNβ ELISA kit (PBL Assay Science) or a Mouse IFNβ ELISA kit (Abcam) and Verikine Mouse IFNα ELISA kit (PBL Assay) or an IFNα Mouse ELISA kit (Invitrogen), respectively. TNF, CXCL1C, GM-CSF, TGFβ1, IL-6, IFNλ and CCL2 levels were assessed by ELISA (R&D Systems). PGE_2_, 12(*S*)-HETE, 15(*S*)-HETE, cysteinyl leukotrienes and LTB_4_ levels were determined by ELISA (Cayman Chemical or Abcam). Surfactant protein-A ELISA was from Cusabio. Cytokines were measured in resting AM supernatant (24 h) using a Proteome Profiler Mouse Cytokine Array Kit, Panel A (R&D Systems) following the manufacturer’s instructions. The pixel intensity was analysed using ImageJ.

### RNA isolation and quantitative PCR with reverse transcription

RNA from AMs, PMs and BMDMs was extracted using a RNeasy kit (Qiagen) according to the manufacturer’s instructions. RNA was reverse transcribed using ABM 5X RT MasterMix (ABM) or LunaScript RT SuperMix (New England Biolabs) as directed by the manufacturer. cDNA was generated by quantitative PCR using BrightGreen Sybr Green (ABM) or PowerUp Sybr Green (Life Technologies). Primers are listed in Supplementary Table 2. C_q_ values obtained using a CFX96 PCR system (Bio-Rad) were analysed using the formula ${2}^{-\Delta {{\rm{C}}}_{{\rm{q}}}}$, normalizing target gene expression to *Gapdh*.

### Adoptive transfer models

AMs from WT or *Alox15*^*−/−*^ mice were collected as described above and resuspended at a density of 5 × 10^4^ cells per 50 µl. AMs were then transferred by the intratracheal route into *Alox15*^*−/−*^ mice (AM populations, IAV infection model) or *Csf2rb*^*−/−*^ mice (BAL protein analysis). For the IAV infection model, mice were infected with IAV PR8 (50 p.f.u., intranasally) 2 h after transfer. BAL and lung tissue were collected and processed as described above for flow cytometry, viral load or total BAL protein content evaluation.

### Administration of 12-HETE or 15-HETE, TG6-10-1, LY255283 or anti-FcεRI and anti-Ly6G antibodies

Exogenous 12-HETE and 15-HETE were purchased from Cayman Chemicals. PND1 and PND2 *Alox15*^*−/−*^ pups or adult mice were intranasally administered with vehicle, 12-HETE or 15-HETE (200 ng in 6 µl or 25 µl of PBS).

TG6-10-1 (5 mg kg^–1^ in 25 µl or 100 µl of PBS) and LY255283 (5 mg kg^–1^ in 25 µl or 100 µl of PBS) were purchased from Cayman Chemicals and intraperitoneally administered to pups (PND0, PND1 or PND2) or adult mice.

Isotype control (rat IgG2a) or anti-Ly6G (50 µg per mouse in 25 µl, both from BioLegend) were given intraperitoneally to PND0, PND1 and PND2 WT pups.

Isotype control (Armenian hamster IgG) or anti-FcεR1 (7 µl of a 100 µg solution per mouse, both from Life Technologies) were given intranasally to WT pups at PND1 and PND2.

### Macrophage proliferation and growth assays

For the macrophage growth assay, 20 × 10^4^ cells were seeded in a 24-well plate and cultured with 20 ng ml^–1^ recombinant murine GM-CSF, 50 ng ml^–1^ M-CSF alone or in combination with IL-4 (20 ng ml^–1^) for 3 days. Cells were then stained with crystal violet, resuspended in ethanol and the optical density read at 595 nm. In some experiments, macrophages were treated with various concentrations of PGE_2_ (1–10 µM, Cayman Chemical).

### Endothelial permeability

Infected or uninfected mice were intraperitoneally injected with 400 µl of Evan’s blue dye (2% in PBS). After 1 h, mice were euthanized, BAL collected and lungs were perfused with 10 ml of PBS. Evan’s blue was then extracted by overnight incubation in formamide at 56 °C (lungs) or overnight incubation with 50% trichloroacetic acid at 4 °C (BAL) and quantified by spectrophotometry analysis using a standard curve of Evan’s blue in formamide or 50% trichloroacetic acid.

### BM chimeras

CD45.1^+^ WT mice or CD45.2^+^ WT or *Alox15*^*−/−*^ mice were lethally irradiated with 9 Gy following 3 days of antibiotic treatment (0.5 g Enrofloxacin (Bayer) per litre of drinking water). After 16 h, the BM compartment was reconstituted with 4 × 10^6^ nucleated cells from either CD45.1^+^ mice (*Alox15*^*−/−*^ or WT CD45.2^+^ recipient) or *Csf2rb*^*−/−*^ or *Alox15*^*−/−*^ mice (*Alox15*^*−/−*^ or CD45.1^+^ recipient) and antibiotic treatment was maintained for 2 additional weeks. Eight weeks after injection, mice were then used for downstream assays.

### AM depletion

WT, *Alox15*^*−/−*^ or *Ccr2*^*−/−*^ mice were treated with control or clodronate liposomes (70 µl, intranasally; Liposoma BV). The AM populations were evaluated at day 2 and day 14 after delivery in the BAL by flow cytometry.

### Extracellular flux analysis

Real-time OCRs of AMs, PMs and BMDMs were measured in XF medium (non-buffered RPMI containing 2 mM l-glutamine, 25 mM glucose and 1 mM sodium pyruvate) using a Seahorse Xfe 96 Analyzer (Agilent Technologies). For the mitochondrial stress test, mitochondrial inhibitors oligomycin (1.5 µM), fluorocarbonyl cyanide phenylhydrazone (FCCP) (1 µM), antimycin A and rotenone (0.5 µM) were used as per the manufacturer’s recommendations. In brief, cells were seeded at a density of 100,000 cells per well and 3 basal measurements were taken. Following this, two consecutive measurements were taken following each injection of oligomycin, FCCP and antimycin A with rotenone. All measurements were normalized to cell number using crystal violet dye extraction assay. Oxygen consumption curves, OCRs and ECARs were generated using Wave Desktop 2.3 (Agilent Technologies).

### Library preparation and RNA-seq

Bulk RNA was collected from BAL AMs from four WT and four *Alox15*^*−/−*^ mice. Sequencing libraries were constructed using the Illumina TruSeq protocol. Libraries were sequenced on an Illumina NovaSeq (paired-end 100 base pair) to an average depth of 42.6 million reads per sample.

### Quantification of gene expression and identification of differential genes

All the reads were mapped to the mouse genome (UCSC mm10) (http://www.ccb.jhu.edu/software/hisat/index.shtml) using HISAT2 (v.2.1.0)^39^ with the default settings. We then used HTSeq-count^40^ to quantify the raw counts for all genes based on the mapped reads using the mm10 gene annotation GTF file downloaded from the UCSC genome browser. With the quantified raw count for all samples, DESeq2 (ref. ^41^) was used to identify differentially expressed genes between samples of the WT and *Alox15* knockout (KO) macrophages in our study. DESeq2 also normalizes gene expression of all samples. The normalized gene expression was further converted into the log_2_ space. We obtained four replicates each from WT (WT0–WT4) and KO (KO1–KO4) macrophage cells. The gene expression pattern of WT4 was different (Mann–Whitney *U-*test *P* = 0) to all other WT samples (WT0–WT3). Therefore we removed WT4 from all subsequent analyses; this decision was supported by the principal component analysis plot (WT4 is far from the other three WT samples in the plot).

We also wrote a JavaScript-based web service to interactively plot the gene expression pattern in different samples and to perform statistical comparison analysis (that is, Mann–Whitney *U*-test) between macrophages in different conditions (for example, WT compared with KO). For any input gene (or gene list), the web service can automatically plot the heatmap (to show gene expression across samples) and violin plot (to show the expression difference between macrophages in different samples and conditions). The normalized gene expression for the queried gene (gene list) can also be directly downloaded from the web service. The web service is freely available at http://junding.lab.mcgill.ca/reseach/maziar/GeneSetEnrichment for easier and interactive use of the RNA-seq datasets generated in this study.

### KEGG Pathway enrichment analyses

We performed pathway enrichment analyses on the identified differential gene list. We first downloaded the mouse pathway annotation from the KEGG database^42^. For a given gene list of interest, we used the hypergeometric test to check whether it was significantly enriched with specific pathways (that is, whether the gene list was significantly enriched with genes from a specific KEGG Pathway). All the obtained *P* values were corrected for multiple comparison.

### Single-cell RNA-seq data generation

Lungs were collected from WT and *Alox15*^*−/−*^ PND1 pups and then the total CD45^+^ cells were isolated using magnetic sorting (StemCell Technologies). Single-cell gel beads in emulsion (GEMs) were generated using a Chromium Controller instrument (10x Genomics). Sequencing libraries were prepared using Chromium Single Cell 3′ Reagent kits (10x Genomics) according to the manufacturer’s instructions. In brief, GEM-RT was performed in a thermal cycler with the following parameters: 53 °C for 45 min; and 85 °C for 5 min. cDNA was cleaned up with DynaBeads MyOne Silane Beads (ThermoFisher Scientific) and amplified with a thermal cycler with the following parameters: 98 °C for 3 min; cycled 12× 98 °C for 15 s; 67 °C for 20 s; 72 °C for 1 min; and 72 °C 1 min. After a clean-up using a SPRIselect Reagent kit, the libraries were constructed by performing the following steps: fragmentation, end repair, A-tailing, SPRIselect cleanup, adaptor ligation, SPRIselect cleanup, sample index PCR and SPRIselect size selection. Libraries were sequenced on a NovaSeq S2 flowcell.

### Single-cell RNA-seq data analyses

Raw fastq files were processed using Cellranger (v.3.1.0)^43^. After filtering for low counts cells using Cellranger’s default parameters, 7,755 and 10,098 cells were retained for WT and *Alox15*^*−/−*^ samples, respectively. The two datasets were integrated using SCTransform^44^. To account for potential variation in cell death and the proliferative status of the cells, we included as covariables the percentage of mitochondrial reads and the expression level of cell cycles genes, respectively. Integration was performed using 3,000 hypervariable genes followed by dimensionality reductions runPCA (retaining 20 principal components for all dependent analysis) and runUMAP. After dimensionality reduction, unsupervised clustering was performed using FindNeighbors and FindClusters (resolution of 1).

Owing to the unprofiled nature of developing mouse lung cells, a combination approach was taken to annotate the different cell type clusters identified. First, we used canonical markers of the different cell types expected to be found in mature lung cells. This was followed by a more systematic cell-type annotation using signature gene sets. Gene sets were derived from the full Panglao cell-type marker database^45^, which were converted to mouse gene nomenclature before use. Annotation was performed using the procedure as described in the R package Cell-ID^46^. In brief, the SCTransform normalized count matrix is evaluated against reference gene lists using a multiclass hypergeometric test. For each cell, the predicted class with the lowest FDR (Benjamini–Hochberg) *P* value was considered the likely true class. *P* values greater than 0.01 were considered ambiguous for this analysis.

To provide more detailed insight, we sought to further characterize neutrophil heterogeneity. To do so, we leveraged a previous a single-cell study^46^ that characterized distinct mouse peripheral blood neutrophil subpopulations through their development. Raw count and per-barcode labels were downloaded from the Gene Expression Omnibus (identifier GSE137539). Counts were log_10_-normalized and then genes were scaled using default parameters of Seurat’s NormalizeData and ScaleData functions. Next, multiple correspondence analysis (MCA and RunMCA function) dimensionality reduction from the Cell-ID package was performed followed by the GetCellGeneSet Cell-ID function. This procedure resulted in a series of custom gene lists (*n* = 200 per list) that defined each neutrophil class. Cell annotation was again performed using these lists to obtain neutrophil subtype predictions. Final labelling was done by assigning unsupervised clusters by their consensus label and manually curating when necessary.

Characterization of differences between WT and *Alox15*^*−/−*^ samples was performed by observing variation in abundance among annotated cell types. We did so using neighbourhood-based differential-abundance testing and visualization with Milo^47^. Because Milo requires at least two samples per condition, each sample was randomly split into two equal ‘pseudosamples’ for use with Milo differential-abundance  testing using the following R function: **≈**condition + pseudosample. Differential-abundance neighbourhood graph construction was completed with the plotNhoodGraphDA.

### ATAC-seq

ATAC-seq was performed on FACS-sorted neonatal (PND3) and BAL-isolated adult alveolar macrophages. Fifty thousand cells were washed once in ice-cold PBS by centrifugation at 500*g* for 5 min at 4 °C. Cells were resuspended in 50 µl lysis buffer (10 mM Tris-HCl, pH 7.4, 10 mM NaCl, 3 mM MgCl_2_ and 0.1% IGEPAL CA-630) and spun down immediately at 500*g* for 10 min at 4 °C. Pellets were resuspended in transposition reaction mix (25 µl 2× TD Buffer (Illumina, FC-121-1030), 2.5 µl Tn5 Transposes (Illumina, FC-121-1030) and 22.5 µl nuclease-free H_2_O) and incubated at 37 °C for 30 min. DNA was purified with a Qiagen MiniElute kit and amplified with Nextera PCR primers (Illumina Nextera Index kit) and NEBNext PCR master mix (M0541, New England BioLabs). Amplified DNA was purified with a Qiagen Mini Elute kit. Libraries were sequenced paired-end on a Novaseq sequencer at the University of Chicago Genomics Facility.

### Initial processing of ATAC-seq data

ATAC-seq reads were mapped to the mouse reference genome (GRCm38/mm10) using Bowtie2. Mapped reads were filtered and sorted using the samtools ‘view’ and ‘sort’, respectively. PCR duplicates were removed using the Picard MarkDuplicates program with parameter REMOVE_DUPLICATES=True. Peaks were subsequently called using the MACS2 software suite^48^ with parameters -q 0.05 and –keep-dup all.

### Differential chromatin accessibility analysis

Differentially accessible peaks were determined first by counting the number of reads overlapping each called peak region (using a merged peak file from all samples and replicates across the time course) using featureCounts software (from the subread package). The resulting count matrix was then further analysed using the limma package for R (v.4.1.1). Features were first filtered by removing those with median log(counts per million) of ≤1 across all samples and replicates. Then we normalized raw counts across all samples using the calcNormFactors function implemented in the R package edgeR (v.3.34.0), which utilizes the TMM algorithm (weighted trimmed mean of *M*-values) to compute normalization factors and we log-transformed the data using the voom function from the limma package (v.3.48.3). After filtering and normalization, we performed a weighted fit with the voom-calculated weights using the lmFit function from limma. The normalized, log-transformed counts were fit to a linear model with the following design: chromatin accessibility **≈**1 + age + genotype:age. This enabled us to capture the effect of *Alox15* KO (genotype) on peak accessibility levels independently for adults and pups. Features with adjusted *P* values smaller than 0.05 were considered significant.

### GSEAs

GSEAs were performed using the R package fgsea (v.1.18.0) with the following parameters: minSize = 15, maxSize = 500, nperm = 100000. To investigate biological pathway enrichments among genes close to differentially accessible peaks, we first assigned all peaks to their nearest gene using the Homer function annotatePeaks with default parameters. Then we ordered genes by their peak associated *t*-statistic derived from limma (see above) and compared the rank-ordered gene list to the reactome gene sets from the MsigDB collections (category = C2, subcategory = CP:REACTOME).

### LPS lung injury

Mice were administered with 20 µg of LPS in PBS (25 µl per mouse, intranasally). BAL and lungs were collected 1 or 3 days after delivery for downstream analyses.

### Intravascular staining

PND1, PND3 or adult WT mice were given 2 µg of FITC-conjugated anti-CD45.2 intravenously to label all circulating cells. Three minutes later, mice were euthanized and lungs collected, stained ex vivo with BUV395-conjugated anti-CD45.2 antibody to determine the parenchymal (cells only labelled with the ex vivo antibody) or vascular localization of the cells (cells labelled with both antibodies).

### SA-β-galactosidase staining

AMs from WT, *Alox15*^*−/−*^*, Alox15*^*lox/lox*^ and *Alox15*^*lox/lox*^*Mrp8*^*cre*^ mice were collected as described above and stained using a colorimetric SA-β-galactosidase staining kit (Cell Signaling Technology) or a CellEvent Senescence kit (Life Technologies) according to manufacturer’s instructions.

### Intravital microscopy

For imaging experiments, male mice were used between 6 and 12 weeks of age. Mice were anaesthetized by an intraperitoneal injection of ketamine (100 mg kg^–1^) and xylazine (10 mg kg^–1^). Anaesthetized mice were cannulated at the internal jugular veins to allow for intravenous injection of antibodies, reagents and additional anaesthetics before and during imaging. Mice were placed on mechanical ventilation through a tracheal catheter. The left lung was exposed by removing two or three ribs. A vacuum chamber with a glass slide fitted on top was used to gently stabilize a portion of the lung for imaging. Anti-Ly6G (clone 1A8, BioLegend) and anti-CD31 (clone MEC13.3, BioLegend) antibodies conjugated with either AlexaFluor 647 or 594 fluorochromes were intravenously injected (7 μl per mouse) to visualize neutrophils and the vasculature, respectively. A resonant-scanner confocal microscope (Leica SP8) was used for all pulmonary imaging. A ×25/0.95 water-objective lens was used for imaging for all videos. A tuneable multiline white light laser was used to simultaneously excite the required fluorochromes. Videos (each 10 min in length) from 3 different field of views were recorded at identical time points and repeated over 2 h of the imaging experiment. Videos were processed using Imaris 9.3. Analysis of cell behaviour and cluster quantification was performed with Imaris 9.3.

### Oil Red O staining

BAL AMs were stained using an Oil Red O staining kit (Abcam) following the manufacturer’s instructions.

### Analysis of pulmonary function

Airway responses to methacholine were evaluated using a small animal ventilator (flexiVent apparatus and flexiVent 5.1 software) as previously described^49^.

### AM phagocytosis assay

BAL phagocytosis was evaluated using pHrodo Green *Escherichia coli* BioParticles Conjugate for Phagocytosis (Invitrogen) following the manufacturer’s instructions. Fluorescence was measured using a Tecan plate reader.

### BAL turbidity

BAL from naive mice were collected as described above, and 200 µl was used to determine the optical density at 600 nm.

### Ethics statement

All experiments involving animals were approved by the McGill University Animal Care Committee (permit number 2010–5860) in accordance with the guidelines set out by the Canadian Council on Animal Care. All animal protocols for intravital lung imaging were approved by the University of Calgary Animal Care Committee (protocol number AC18-0038).

### Statistical analysis

Data are presented as the mean ± s.e.m. Statistical analyses were performed using GraphPad Prism v.9.1.2 software (GraphPad). Statistical differences were determined using two-sided log-rank test (survival studies), one-way analysis of variance (ANOVA) followed by Tukey’s multiple comparisons test, two-way ANOVA followed by Sidak’s or Tukey’s multiple comparisons test, paired or unpaired two-tailed *t*-test or two-tailed Mann–Whitney test.

### Reporting Summary

Further information on research design is available in the Nature Portfolio Reporting Summary linked to this article.

## Online content

Any methods, additional references, Nature Portfolio reporting summaries, source data, extended data, supplementary information, acknowledgements, peer review information; details of author contributions and competing interests; and statements of data and code availability are available at 10.1038/s41586-022-05660-7.

## Supplementary information

Supplementary Figure 1 Flow cytometry gating strategies for the evaluation of immune cells in the lungs of naive adult (6–8 weeks) WT or *Alox15*^*–*^^*/–*^ mice. **a**, Cells were gated on single live CD45.2^+^ cells then further gated on CD11c^+^ and Siglec-F^+^ (AMs), CD11c^–^Siglec-F^–^Ly6G^+^CD11b^+^ (neutrophils), Ly6G^–^CD11b^+^F4/80^+^Ly6C^+/–^ for monocytes/macrophages. **b**, Natural killer cells were gated on single live cells, CD64^–^Ly6G^–^NK1.1^+^. **c**, Conventional dendritic cells were gated on single live cells, CD64^–^Ly6C^–^CD11c^+^, CD11b^–^CD103^+^ (cDC1) or CD11b^+^CD103^–^ (cDC2).

Reporting Summary

Supplementary Table 1 List of differentially expressed genes in adult WT and *Alox15*^*–/–*^ AM bulk RNA-seq dataset.

Supplementary Table 2 List of primers used in this study.

Supplementary Video 1 Pulmonary intravital microscopy was used to compare the effects of intravenous LPS (20 µg intravenously) in WT or *Alox15*^*–/–*^ mice. Neutrophils (cyan) are labelled with AlexaFluor-647 anti-Ly6G antibody and the vascular endothelium (red) is labelled with AlexaFluor-594 conjugated anti-CD31 antibody. A large neutrophil cluster is demonstrated in the second part of the movie with an arrow.

## Data Availability

All data supporting the findings of this study are included in the published article and supplementary materials. Bulk RNA-seq, ATAC-seq and single-cell RNA-seq data have been deposited into the Gene Expression Omnibus and are publicly available under accession numbers GSE216531 and GSE219042. Source data are provided with this paper.

## Source data

Source Data Fig. 1
Source Data Fig. 2
Source Data Fig. 3
Source Data Fig. 4
Source Data Fig. 5
Source Data Extended Data Fig. 1
Source Data Extended Data Fig. 2
Source Data Extended Data Fig. 3
Source Data Extended Data Fig. 4
Source Data Extended Data Fig. 5
Source Data Extended Data Fig. 6
Source Data Extended Data Fig. 7
Source Data Extended Data Fig. 8
Source Data Extended Data Fig. 9
Source Data Extended Data Fig. 10
