# Supplementary material for: Neonatal imprinting of alveolar macrophages via neutrophil-derived 12-HETE
Source: Nature. 2023 Jan 4;614(7948):530–8. doi: 10.1038/s41586-022-05660-7 (PMC9945843; doi:10.1038/s41586-022-05660-7)

nature portfolio

https://doi.org/10.1038/s41586-022-05660-7

Supplementary information

Neonatal imprinting of alveolar macrophages via neutrophil-derived 12-HETE

In the format provided by the authors and unedited

Supplementary Figure 1

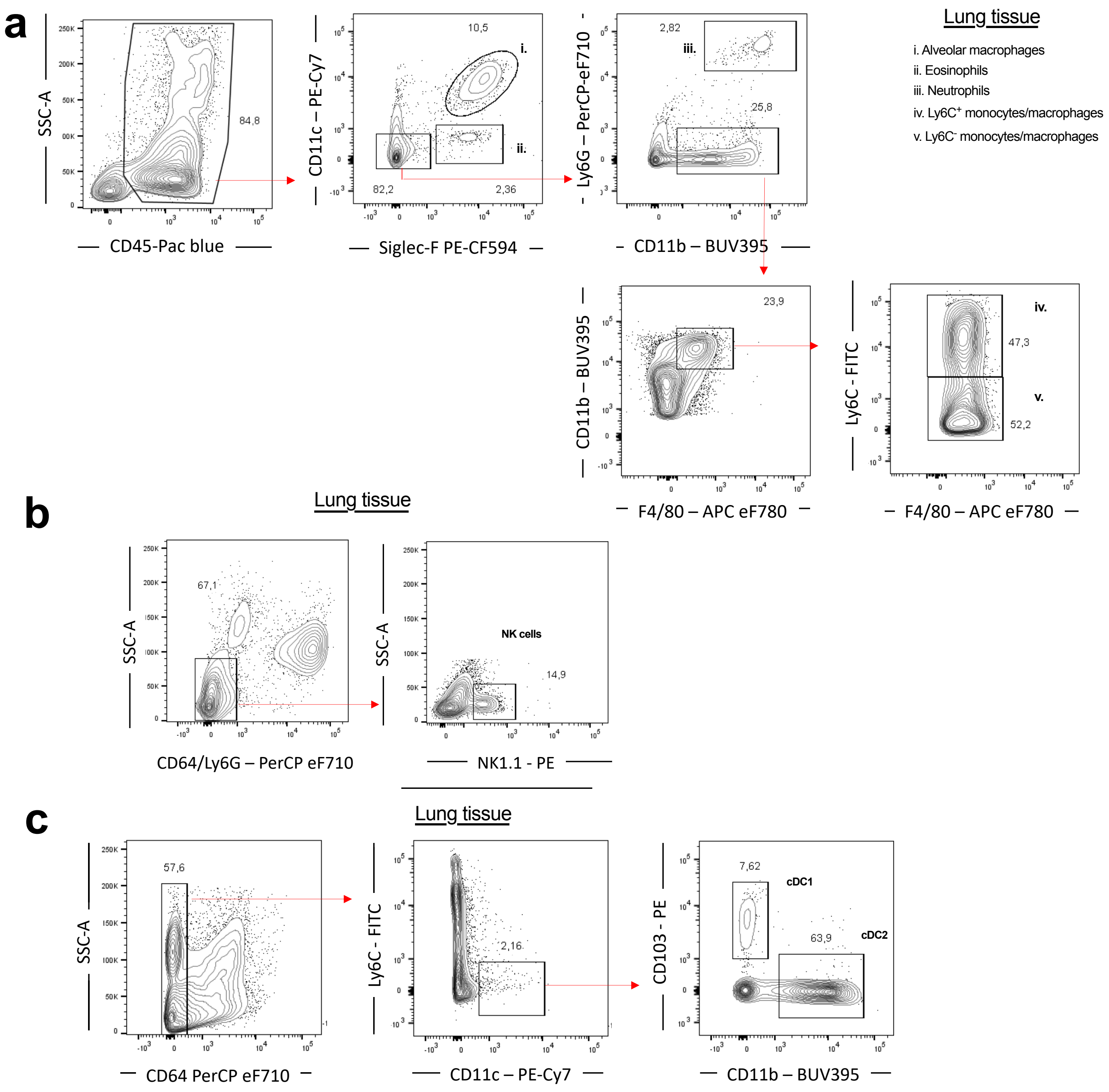

Supplement: Supplementary file 1 — Flow cytometry gating strategies for the evaluation of immune cells in the lungs of naive adult (6–8 weeks) WT or Alox15–/– mice. a, Cells were gated on single live CD45.2+ cells then further gated on CD11c+ and Siglec-F+ (AMs), CD11c–Siglec-F–Ly6G+CD11b+ (neutrophils), Ly6G–CD11b+F4/80+Ly6C+/– for monocytes/macrophages. b, Natural killer cells were gated on single live cells, CD64–Ly6G–NK1.1+. c, Conventional dendritic cells were gated on single live cells, CD64–Ly6C–CD11c+, CD11b–CD103+ (cDC1) or CD11b+CD103– (cDC2). [file 41586_2022_5660_MOESM1_ESM.pdf]
