# Supplementary material for: Neonatal imprinting of alveolar macrophages via neutrophil-derived 12-HETE
Source: Nature. 2023 Jan 4;614(7948):530–8. doi: 10.1038/s41586-022-05660-7 (PMC9945843; doi:10.1038/s41586-022-05660-7)
Supplement: Supplementary file 2 — Reporting Summary [file 41586_2022_5660_MOESM2_ESM.pdf]

nature portfolio

Corresponding author(s): Dr. Maziar Divangahi

Last updated by author(s): Nov 15, 2022

Reporting Summary

Nature Portfolio wishes to improve the reproducibility of the work that we publish. This form provides structure for consistency and transparency in reporting. For further information on Nature Portfolio policies, see our Editorial Policies and the Editorial Policy Checklist.

Statistics

For all statistical analyses, confirm that the following items are present in the figure legend, table legend, main text, or Methods section.

n/a	Confirmed	
☐	☒	The exact sample size (*n*) for each experimental group/condition, given as a discrete number and unit of measurement
☐	☒	A statement on whether measurements were taken from distinct samples or whether the same sample was measured repeatedly
☐	☒	The statistical test(s) used AND whether they are one- or two-sided<br>*Only common tests should be described solely by name; describe more complex techniques in the Methods section.*
☐	☒	A description of all covariates tested
☐	☒	A description of any assumptions or corrections, such as tests of normality and adjustment for multiple comparisons
☐	☒	A full description of the statistical parameters including central tendency (e.g. means) or other basic estimates (e.g. regression coefficient) AND variation (e.g. standard deviation) or associated estimates of uncertainty (e.g. confidence intervals)
☐	☒	For null hypothesis testing, the test statistic (e.g. *F*, *t*, *r*) with confidence intervals, effect sizes, degrees of freedom and *P* value noted<br>*Give P values as exact values whenever suitable.*
☒	☐	For Bayesian analysis, information on the choice of priors and Markov chain Monte Carlo settings
☒	☐	For hierarchical and complex designs, identification of the appropriate level for tests and full reporting of outcomes
☐	☒	Estimates of effect sizes (e.g. Cohen's *d*, Pearson's *r*), indicating how they were calculated

Our web collection on statistics for biologists contains articles on many of the points above.

Software and code

Policy information about availability of computer code

Data collection	BD FACS Diva v8.0.1, Zeiss Zen v2.3 SP1 FP1, Imaris 9.3, Wave desktop v2.3
Data analysis	TreeStar Flowjo V10.8, Graphpad Prism v7.05, ImageJ v1.51, Zeiss Zen v2.3 SP1 FP1, Wave desktop v2.3, HISAT2 v2.1, DESeq2, CellRanger v3.1.0

For manuscripts utilizing custom algorithms or software that are central to the research but not yet described in published literature, software must be made available to editors and reviewers. We strongly encourage code deposition in a community repository (e.g. GitHub). See the Nature Portfolio guidelines for submitting code & software for further information.

Data

Policy information about availability of data

All manuscripts must include a data availability statement. This statement should provide the following information, where applicable:

- Accession codes, unique identifiers, or web links for publicly available datasets
- A description of any restrictions on data availability
- For clinical datasets or third party data, please ensure that the statement adheres to our policy

All data supporting the findings of this study are included in the published article and supplementary materials. Bulk RNA-Sequencing, ATAC-Sequencing and single-cell RNA-Sequencing have been deposited in Gene Expression Omnibus (GEO) and are publicly available under accession numbers GSE216531 and GSEXXXX. Source data are provided with this paper.

Field-specific reporting

Please select the one below that is the best fit for your research. If you are not sure, read the appropriate sections before making your selection.

☒ Life sciences ☐ Behavioural & social sciences ☐ Ecological, evolutionary & environmental sciences

For a reference copy of the document with all sections, see nature.com/documents/nr-reporting-summary-flat.pdf

Life sciences study design

All studies must disclose on these points even when the disclosure is negative.

Sample size	Sample sizes were empirically determined to optimize numbers based on our previous experience with equivalent experiments. In most of the experiments, 3 to 12 mice/group/ timepoint was sufficient to identify differences between groups with a 5% significance level.
Data exclusions	In the RNA-Seq dataset, we have 4 replicates from WT (WT0-WT4) and KO (KO1-KO4) macrophage cells, respectively. The gene expression pattern of WT4 is very different (Mann Whitney U test p-value=0) to all other WT samples (WT0-WT3) and thus we removed WT4 from all following analyses, which is also supported by the PCA plot (WT4 is far from all other 3 WT samples in the PCA plot).
Replication	All attempts at replicating the results were successful. The number of biological replicates and experiments are provided in the figure legends
Randomization	Samples/mice were randomly allocated to different groups.
Blinding	Investigators were not blinded because each experiment was performed by a single researcher.

Reporting for specific materials, systems and methods

We require information from authors about some types of materials, experimental systems and methods used in many studies. Here, indicate whether each material, system or method listed is relevant to your study. If you are not sure if a list item applies to your research, read the appropriate section before selecting a response.

Materials & experimental systems

n/a	Involved in the study	
☐	☒	Antibodies
☐	☒	Eukaryotic cell lines
☒	☐	Palaeontology and archaeology
☐	☒	Animals and other organisms
☒	☐	Human research participants
☒	☐	Clinical data
☒	☐	Dual use research of concern

Methods

n/a	Involved in the study	
☒	☐	ChIP-seq
☐	☒	Flow cytometry
☒	☐	MRI-based neuroimaging

Antibodies

Antibodies used

Viability dye e450 or e506 (Invitrogen) (cat#65-0863-18 and 65-0866-18, 1/1000)
Anti-CD16/32 (BD Bioscience) (cat#553142, clone 2.4G2, 1/200)
PE-Cy7- or FITC-conjugated anti-CD11c (BD Bioscience) (cat#558079 and 553801, clone HL3, 1/200)
PE-CF594- or BV786-conjugated anti-SiglecF (BD Bioscience) (cat#562757 and 740956, clone E50-2440, 1/200)
PE-Cy7- or BUV395-conjugated anti-CD11b (BD Bioscience) (cat#561098 and 563553, clone M1/70, 1/200)
PerCP-eFluor710-conjugated anti-Ly6G (Invitrogen) (cat#46-9668-82, clone 1A8, 1/200)
APC-conjugated anti-Ly6G (BD Biosciences) (cat#560599, clone 1A8, 1/200)
APC-conjugated anti-Ly6C (Invitrogen) (cat#17-5932-82, clone HK1.4, 1/200)
FITC-conjugated anti-Ly6C (BD Biosciences) (cat#553104, clone AL-21, 1/200)
APC-eFluor780- or PE-conjugated anti-F4/80 (Invitrogen) (cat#12-4801-82 and 47-4801-82, clone BM8, 1/100)
PE-conjugated anti-CD103 (BD Bioscience) (cat#557495, clone M290, 1/200)
PerCP-eFluor710-conjugated anti-CD64 (Invitrogen) (cat#46-0641-82, clone X54-5/7.1, 1/200)
BUV395-conjugated anti CD45.2 (BD Bioscience) (cat#564616, clone 104, 1/200)
APC-conjugated anti CD45.1 (BD Bioscience) (cat#558701, clone A20, 1/200)
efluor660-conjugated anti CD68 (Invitrogen) (cat#50-0681-82, clone FA-11, 1/200)
BV785-conjugated anti CX3CR1 (Biolegend) (cat#149029, clone SA011F11, 1/200)
APC- or FITC-conjugated anti-BrdU set (BD Bioscience) (cat#556028 and 552598, 1/50)
e660- or PE-conjugated anti-Ki67 (Invitrogen) (cat#50-5698-82 and 12-5698-92, clone SolA15, 1/400)
PE-conjugated anti-p53 set (BD Bioscience) (cat#557027, 20μl/test)
AlexaFluor647-conjugated anti-p-p38 (BD Bioscience) (cat#612595, clone 36/p38, 20μl/test)
PE-conjugated anti-pSMAD2/3 (BD Bioscience) (cat#562586, clone O72-670, 5μl/test)
APC-conjugated anti-pAKT1 (Invitrogen) (cat#17-9715-42, clone SDRNR, 5μl/test)

PerCP-efluor710-cojugated anti-pERK1/2 (Invitrogen) (cat#46-9109-42, clone MILAN8R, 5µl/test)
PE-Cy7-conjugated anti-p-γH2AX (Biolegend) (cat#613419, clone 2F3, 5µl/test)
APC-conjugated anti-pSTAT5 (Invitrogen) (cat#17-9010-42, clone SRBCZX, 5µl/test)
Alexafluor 647-conjugated anti-15-lipoxygenase 1 (cat#bs-6505R-A647, polyclonal, 1/50)
Purified anti-BrdU (Biolegend) (cat#364101, clone 3D4, 1µg/100µl)
Alexafluor 647-conjugated anti-CD11c (Biolegend) (cat#117312, clone N418)
Alexafluor 594-conjugated anti-Ly6G (Biolegend) (cat#127636, clone 1A8)
Alexafluor 488-conjugated anti-CD31 (Biolegend) (cat#102414, clone 390)
Ultraleaf anti-mouse Ly6G antibody (Biolegend) (cat#127649, clone 1A8, 50µg/mouse)
Ultraleaf purified rat IgG2a Isotype control (Biolegend) (cat#400565, clone RTK2758, 50µg/mouse)
FceR1alpha Monoclonal antibody (Invitrogen) (cat#14-5898-82, clone MAR-1, 7µl/mouse)
Armenian hamster IgG Isotype control (Invitrogen) (cat#14-4888-81, clone eBio299Arm, 7µl/mouse)
PE Annexin V Apoptosis detection kit (BD Bioscience) (cat#559763, 5µl/test)
phospho-STAT5 (Tyr694) rabbit antibody (Cell Signaling Technologies) (cat#4322, clone D47E7, 1/100)
Alexafluor 647-conjugated anti-rabbit IgG (Invitrogen) (cat#A-21235, 1/1000)
Alexafluor 555-conjugated anti-rabbit IgG (Invitrogen) (cat#A-21428, 1/1000)
Alexafluor 647-conjugated anti-mouse IgG (Invitrogen) (cat#A-21235, 1/1000)
Alexafluor 555-conjugated anti-mouse IgG (Invitrogen) (cat#A-21422, 1/1000)
Alexafluor 647-conjugated anti-mouse Ly6G (Biolegend) (cat#127610, clone 1A8, 7µl/mouse)
Alexafluor 594-conjugated anti-mouse CD31 (Biolegend) (cat#102520, clone MEC13.3, 7µl/mouse)

Validation

Antibodies are quality checked and validated by the respective manufacturers and informations regarding the validation can be found on the companies' websites.

Eukaryotic cell lines

Policy information about cell lines

Cell line source(s)

VeroE6, MDCK and L929 cells were from ATCC

Authentication

Growth rate and morphology of cells were checked frequently, but no profiling was performed

Mycoplasma contamination

Cell lines were certified negative for mycoplasma contamination when bought and randomly tested using commercially available kits.

Commonly misidentified lines
(See ICLAC register)

No commonly misidentified cell line were used

Animals and other organisms

Policy information about studies involving animals; ARRIVE guidelines recommended for reporting animal research

Laboratory animals

Various age (PND0, PND1, PND3, 3 weeks, 6-52 weeks) mice were used in this study. Strains used are C57BL/6 mice, CD45.1 mice, Alox15-/- mice, Csf2rb-/- mice, Alox5-/- mice, MRP8-Cre mice and k18-hACE2 mice and were purchased from Jackson Laboratories. k18-hACE2 Alox15-/- or Alox15+/+ were generated inhouse by crossing Alox15-/- mice with k18-hACE2 mice. Alox15-lox/lox mice were from Dr. Sarah Tersey (University of Chicago) and Ltb4r2-/- mice were from Dr. Charles Brown (University of Missouri). All animals were housed and inbred at the animal facility of the Research Institute of McGill University under SPF conditions with ad libitum access to food and water, temperature of 21ºC (+/- 1ºC), relative humidity of 40-60%RH (+/- 5%RH) and light cycle of 12 hours ON, 12 hours OFF (daily cycle). Experiments were performed using female and male age- and sex-matched mice.

Wild animals

No wild animals were used in this study.

Field-collected samples

No field-collected samples were used in this study.

Ethics oversight

All experiments involving animals were approved by the McGill University Animal Care Committee (Permit # 2010-5860) in accordance with the guidelines set out by the Canadian Council on Animal Care.
All animal protocols for intravital lung imaging were approved by the University of Calgary Animal Care Committee (Protocol #: AC18-0038).

Note that full information on the approval of the study protocol must also be provided in the manuscript.

Flow Cytometry

Plots

Confirm that:

☒ The axis labels state the marker and fluorochrome used (e.g. CD4-FITC).

☒ The axis scales are clearly visible. Include numbers along axes only for bottom left plot of group (a 'group' is an analysis of identical markers).

☒ All plots are contour plots with outliers or pseudocolor plots.

☒ A numerical value for number of cells or percentage (with statistics) is provided.

Methodology

Sample preparation	Lung tissues were perfused with 10mL of PBS, harvested and minced before collagenase IV digestion (150U/mL, Sigma) for 1 hour at 37C. Lungs were filtered through a 70µm nylon mesh and red blood cells were lysed. Peritoneal cells were obtained following lavage with 5ml of cold PBS injected ip. Cells were then spun down, and red blood cells lysed. Spleen cells were obtained by crushing the spleen on a 70µm nylon mesh followed by red blood cells lysis. Liver cells were obtained after mincing and digestion with collagenase VIII (1mg/ml, Sigma) for 30min at 37C. The cells were passed sequentially through 100 and 70µm cell strainers before red blood cell lysis. Brain cells were obtained after passing through 100 and 70µm cell strainers followed by Percoll gradient (30% and 70% solutions). Total lung, peritoneal, liver, brain and spleen cell counts were determined with a hemocytometer and 0.5-2 million cells were used for staining.
Instrument	Samples were acquired on a BD LSR Fortessa X-20
Software	Samples were collected using BD FACS Diva v8.0.1 and analyzed in Flow Jo v10.8 (TreeStar)
Cell population abundance	The cell populations were sufficient for downstream analysis
Gating strategy	Gating strategies are provided in supplementary figure 1.

☒ Tick this box to confirm that a figure exemplifying the gating strategy is provided in the Supplementary Information.
